# Metabolomic Profiling of Endophytic Fungi and the Host Plant *Annona jahnii* Saff. Reveals Shared and Analogous Compounds

**DOI:** 10.3390/plants15030501

**Published:** 2026-02-05

**Authors:** Luciana Araújo Xavier, Cecília Maria Bezerra de Araújo, Gilmar Prado de Sousa, Eduardo Jorge Pilau, Carla Porto, Antonia Queiroz Lima de Souza, Edineide Cristina Alexandre de Souza, Adriana Flach, Luiz Antonio Mendonça Alves da Costa

**Affiliations:** 1 Programa de Pós-Graduação em Biodiversidade e Biotecnologia da Amazônia, Núcleo de Pesquisas Energéticas, Departamento de Química, Universidade Federal de Roraima, Boa Vista 69310-000, RR, Brazil; luciana.xavier@ufrr.br (L.A.X.); edineide.souza@ufrr.br (E.C.A.d.S.); luiz.costa@ufrr.br (L.A.M.A.d.C.); 2 Departamento de Química, Universidade Federal de Roraima, Boa Vista 69310-000, RR, Brazil; cissa.b.a@hotmail.com; 3 Programa de Pós-Graduação em Química, Universidade Federal de Roraima, Boa Vista 69310-000, RR, Brazil; gilmarprado.sousa@gmail.com; 4 Departamento de Química, Universidade Estadual de Maringá, Maringá 87020-900, PR, Brazil; ejpilau@uem.br; 5 MS Bioscience, Incubadora Tecnológica de Maringá, Complexo UEM, Maringá 87029-900, PR, Brazil; carlaporto@msbioscience.com.br; 6 Programa de Pós-Graduação em Biodiversidade e Biotecnologia da Amazônia, Universidade Federal do Amazonas, Manaus 69067-005, AM, Brazil

**Keywords:** GNPS, molecular networking, *Penicillium*, plant/fungus metabolites

## Abstract

Endophytic fungi are a viable option for obtaining metabolites identical or analogous to those produced by the host plant. However, research on the ability of these microorganisms to biosynthesize these metabolites is still scarce, although important to enable their use for this purpose, contributing to the preservation of the host plant. The metabolomic study of fungal (*Penicillium sumatraense*, *Penicillium miczynskii*, *Penicillium osmophilum*, and *Penicillium chermesinum*) and plant extracts was carried out using UHPLC/ESI-MS/MS analyses combined with exploratory analysis by Molecular Networking (MN). Antioxidant activity by the 1,1-diphenyl-2-picrylhydrazyl (DPPH) free radical method was performed on fungal and plant extracts. The exploratory analysis by MN showed 75 MS features that were detected in the fungi and the host plant; of these, four compounds were putatively identified. The analysis showed 539 MS features with structural similarity to both biological matrices. Fungal extracts showed more promising antioxidant activities when compared to the plant extract. UHPLC combined with Molecular Networking proved to be a powerful strategy to guide the identification of microorganisms capable of biosynthesizing metabolites produced by the host plant. The strategy allowed for an early and efficient evaluation of crude extracts and provided unprecedented information regarding the chemical profile of *A. jahnii* and its endophytic fungi.

## 1. Introduction

Plants are the main resource for obtaining the bioactive metabolites applied in different areas, mainly in the production of medicines [1,2,3]. However, according to Kumar et al., 2013 [4], this extensive demand for bioactive metabolites cannot be met exclusively by exploiting plants, which are a resource susceptible to extinction. In this context, endophytic fungi represent a remarkable alternative for obtaining biologically active compounds [5,6]. The study of these microorganisms can enable the preservation of the host plant, in addition to presenting advantages that include the following: ease of cultivation in fermentation tanks, which provides an unlimited supply of metabolites; obtaining more potent compounds derived from bioactive compounds; simple extraction procedure; and less solvent to purify the final product [4].

Endophytic fungi are able to adapt through prolonged association with their host plants. This adaptation likely results in the broad biosynthetic potential of these microorganisms, exhibiting a metabolic profile strongly influenced by the plant environment [7,8,9]. The host–endophyte interaction also allows endophytic fungi to biosynthesize secondary metabolites that are identical and similar to those obtained by their host [7,10,11].

The exploration of endophytic fungi for the obtention of bioactive metabolites found in plants is promising, but currently limited, mainly by the attenuation or neutralization of the biosynthesis of some of these compounds. The degradation of this biosynthetic capacity is observed in successive fungi cultures, cultivated in isolation in the laboratory, and must be a result of the lack of host stimuli [12,13,14,15]. To overcome this challenge, studies focus on tracking endophytic fungi with a high productive capacity of plant-derived metabolites, in addition to improving this capacity, through genetic engineering studies and the identification of specific elicitors, among others [16]. Research in this area mainly addresses the identification of one or a few known compounds, initially isolated from plants [17,18,19,20,21]. This approach is effective, but limits the discovery of other plant-/fungus-derived compounds and analogs resulting from fungal biotransformation that may present a promising potential as bioactive metabolites [22].

More detailed information on the biosynthetic potential of plant metabolites by endophytic fungi and especially analogous compounds could be obtained from comparative studies of the chemical profile of endophytic fungi and their host plant. Xu et al., 2009 [23], analyzed fungal and host plant extracts using gas chromatography–mass spectrometry. The study enabled the discovery of an attractive bioactive compound obtained in both extracts, in addition to presenting relevant results regarding the biological activities of the fungal extract, suggesting the potential of the endophyte as an alternative resource for obtaining bioactive compounds.

Thus, given the need for rapid and effective strategies that contribute to the development of research in this area, we propose an exploratory analysis of the total metabolomic profile of fungal extracts and the host plant *A. jahnii*, using Molecular Networking (MN) through the GNPS (Global Natural Products Social Molecular Networking) platform. This technique is widely used for the analysis of complex extracts with large volumes of data [24], in addition to allowing the dereplication of known compounds and detection of corresponding analogs or compounds belonging to the same general class of molecules [25]. Therefore, the objective of this study is to provide unprecedented information on the chemical profile of the plant *A. jahnii* and of four isolated endophytic fungi, and to compare the metabolomic profiles of these extracts. It also aims to determine the applicability of MN in guiding the discovery of endophytic fungi capable of biosynthesizing compounds produced by their host plant and analogous compounds.

## 2. Results

### 2.1. Metabolomic Profile of the Extracts

An exploratory analysis of the metabolomic profiles of the crude extracts was performed through the study of spectral data obtained by UHPLC/ESI-MS/MS and transferred to the GNPS platform for the obtention of MN. The datasets generated and/or analyzed during this study are available in the Massive repository, MSV000100358, at the following link: ftp://massive-ftp.ucsd.edu/v11/MSV000100358/, accessed on 30 December 2025.

The MS/MS Molecular Network of the crude extracts resulted in 1263 nodes that were not grouped, once they did not show spectral similarity with any other, in addition to 1441 nodes distributed in 241 clusters, which show plant–plant, fungus–fungus, or plant–fungus groupings. The analysis of the data obtained by MN combined with in silico fragmentation by the MetFrag platform allowed the dereplication of 62 metabolites (Table S1), provisionally annotated with a confidence level of 3 in accordance with the classification proposed by Schrimpe-Rutledge et al., 2016 [26], based on a search in the libraries provided by the two platforms. The results obtained for the metabolomic profile of the combinatorial analysis of the plant and fungal matrix are described in detail below.

#### Molecular Networking for the Comparative Study of the Metabolic Profile of Fungal Extracts and the Host Plant

**Figure 1:**
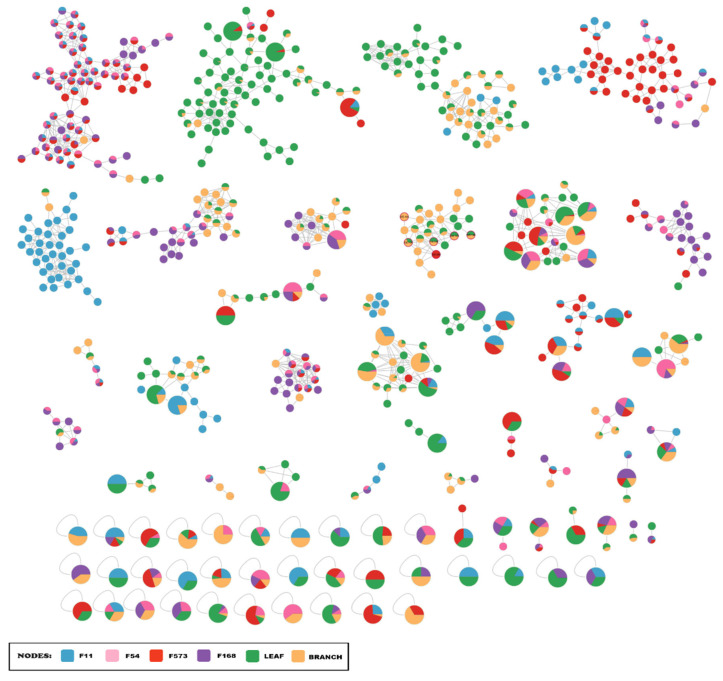
Molecular Networking containing identical and structurally related chemical entities, detected in fungal and plant extracts.

The combination of spectral data from fungal and plant extracts generated a molecular networking with 2704 molecular features, of which 1043 are exclusive to plant extracts and 1586 to fungal extracts. Figure 1 shows the MN containing only the identical and structurally related molecular features in both biological matrices, totaling 614 molecular features, of which 75 were detected in both plant and fungal matrices, represented by the larger nodes in the MN.

**Figure 2:**
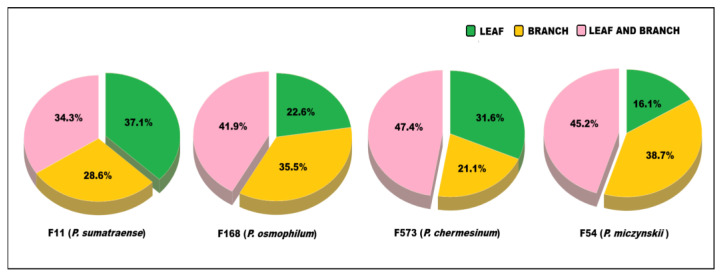
Percentage of shared molecular features between the extracts of each fungal strain and the plant extracts from the leaf and branch of *A. jahnii*.

Exploratory MN analysis allowed the identification of each fungal strain’s ability to biosynthesize the compounds present in the host plant extracts. Figure 2 shows the percentage of molecular features detected in each fungal extract that were also identified in the plant extracts, highlighting the degree of chemical similarity between the metabolic profiles of endophytic fungi and the host plant. Of the total molecular characteristics detected in the plant and fungal matrices, approximately 34.7% were detected in the fungal extracts and branch extracts. Fungi shared 29.3% with leaf extracts and 36% with both plant extracts. The F573 strain showed the highest number of molecular characteristics shared with the plant matrix, totaling 38, of which 47.4% were identified in both the leaf and branch extracts of the host plant. Strains F54 and F168 shared 31 molecular characteristics with the plant extracts, with over 40% of compounds also detected in the leaf and branch fractions. Strain F11, with 35 shared molecular characteristics, stood out from the others by presenting a higher proportion of molecular characteristics also found in the leaf extract. These data demonstrate the ability of both endophytic fungi and the host plant to biosynthesize the same secondary metabolites.

### 2.2. Compounds Detected in Plant and Fungal Extracts and Analogous Compounds

The workflow proposed here for the analysis of crude extracts enabled the putative identification of metabolites from the phenylpropanoid and sesquiterpene classes, detected in both biological matrices, as well as metabolites analogous to plant and fungal extracts.

#### 2.2.1. Phenylpropanoids

**Figure 3:**
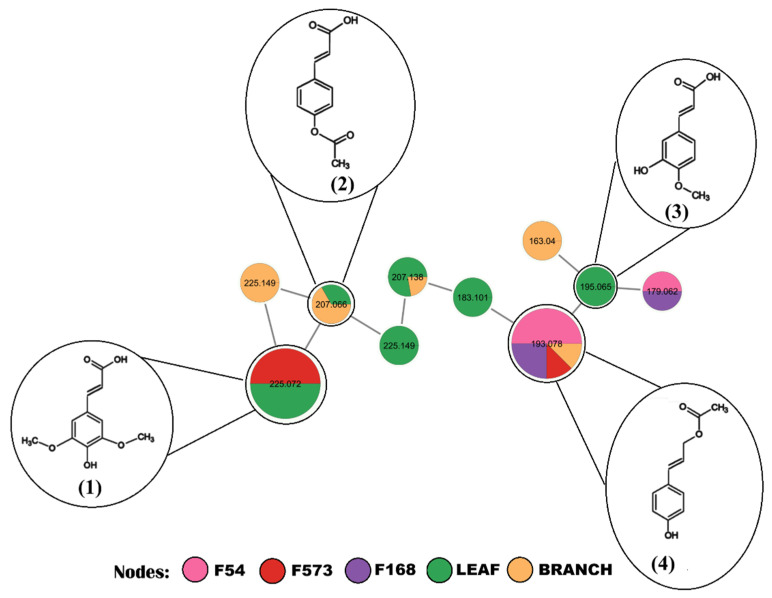
Cluster containing phenylpropanoids putatively identified in fungal (F54—*P. miczynskii*; F168—*P. osmophilum*; F573—*P. chermesinum*) and plant extracts. (1) sinapic acid; (2) 4acetoxycinnamic acid; (3) isoferulic acid; (4) coumaryl acetate.

Two metabolites from the phenylpropanoid class were putatively identified in both fungal and plant extracts. The ions [M + H]^+^, *m*/*z* 193.0855, and [M + H]^+^, *m*/*z* 225.0750 (Figure 3), were putatively identified as coumaryl acetate and sinapic acid, respectively. These metabolites share a common substructure and are therefore interconnected in the same cluster, which also has one molecular characteristic detected in fungal extracts and seven in plant extracts (Figure 3). Sinapic acid was annotated using the similarity score in the GNPS library, which is based on the comparison between experimentally obtained MS/MS spectra and spectra from the GNPS database.

The identification propagation strategy applied to the phenylpropanoid cluster allowed the annotation of coumaryl acetate, produced in three fungal extracts, F54, F168, and F573, and in the branch extract of *A. jahnii*. The other two putatively identified metabolites are 4-acetoxycinnamic acid and isoferulic acid, detected in the plant extracts. The cluster also has five molecular features of plant extracts and one of fungal extracts that represent analogs, that is, compounds with a structure similar to the metabolites obtained in both biological matrices.

**Figure 4:**
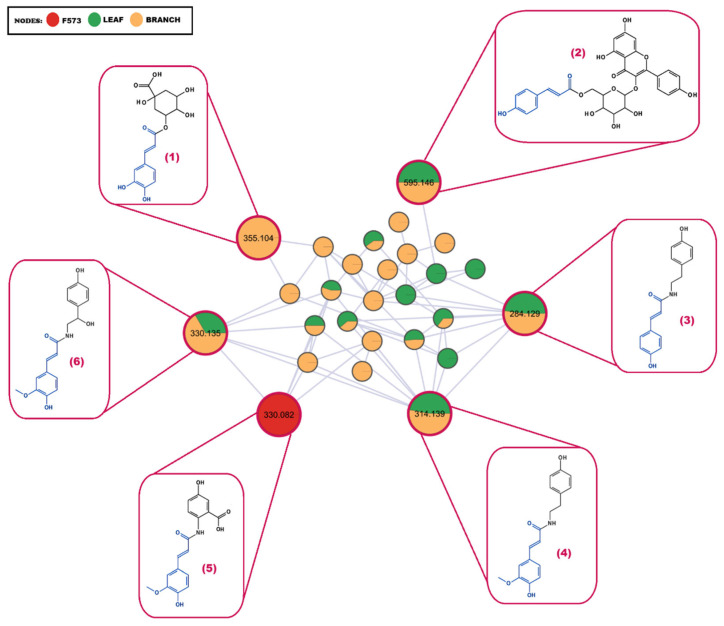
Five metabolites were putatively identified in plant extracts and one metabolite was putatively identified in a fungal extract (F573—*P. chermesinum*), all belonging to the same cluster. (1) Chlorogenic acid (*m*/*z* 355.104 ([M + H]^+^)); (2) Tiliroside (*m*/*z* 595.146 ([M + H]^+^)); (3) Paprazine (*m*/*z* 284.129 ([M + H]^+^)); (4) Feruloyltyramine (*m*/*z* 314.139 ([M + H]^+^)); (5) Avenanthramide B (*m*/*z* 330.082 ([M + H]^+^)); and (6) *N*-*trans*-Feruloyloctopamine (*m*/*z* 330.135 ([M + H]^+^)).

The proposed workflow within the MN allowed the dereplication of other fungal metabolites analogous to plant-derived compounds belonging to the phenylpropanoid class. Figure 4 shows six compounds belonging to the same cluster and putatively identified. The metabolites paprazine ([M + H]^+^, *m*/*z* 284.1280), feruloyltyramine ([M + H]^+^, *m*/*z* 314.1384), chlorogenic acid ([M + H]^+^, *m*/*z* 355.1019), N-trans-feruloyloctopamine ([M + H]^+^, *m*/*z* 330.1331), and tiliroside ([M + H]^+^, *m*/*z* 595.1439) are present in the plant extracts. The [M + H]^+^ ion, *m*/*z* 330.0952, putatively identified as avenanthramide B, was detected in the fungal extract F573. The metabolites proposed by the GNPS library for each ion were also the best-ranked candidates in the in silico fragmentation obtained on the Metfrag platform. These results reinforce the probability that the candidates proposed in the putative identification actually represent the metabolites present in each biological matrix.

The ion putatively identified as chlorogenic acid, which has already been isolated from *Annona* species [27,28], presented a fragment ion with *m*/*z* 163 corresponding to the [C_9_H_7_O_3_]^+^ unit, highlighted in blue (Figure 4). The avenanthramide B present in the fungal extract is structurally related to feruloyltyramine and N-trans-feruloyloctopamine; these metabolites share the same fragment ions with *m*/*z* 177 and *m*/*z* 145, corresponding to the [C_10_H_9_O_3_]^+^ and [C_9_H_6_O_2_-H]^+^ units, respectively. The portion highlighted in blue in the structural formula of each metabolite, avenanthramide B, feruloyltyramine, and *N-trans*-feruloyloctopamine, is structurally identical and similar to that present in the other three putatively identified metabolites. Chlorogenic acid has two hydroxyl groups on the benzene ring, while the portion highlighted in blue of paprazine and tiliroside has only one hydroxyl group. Tiliroside has the highest mass charge of the cluster, a characteristic assigned to the glycosylated flavonoid portion.

#### 2.2.2. Sesquiterpenes

**Figure 5:**
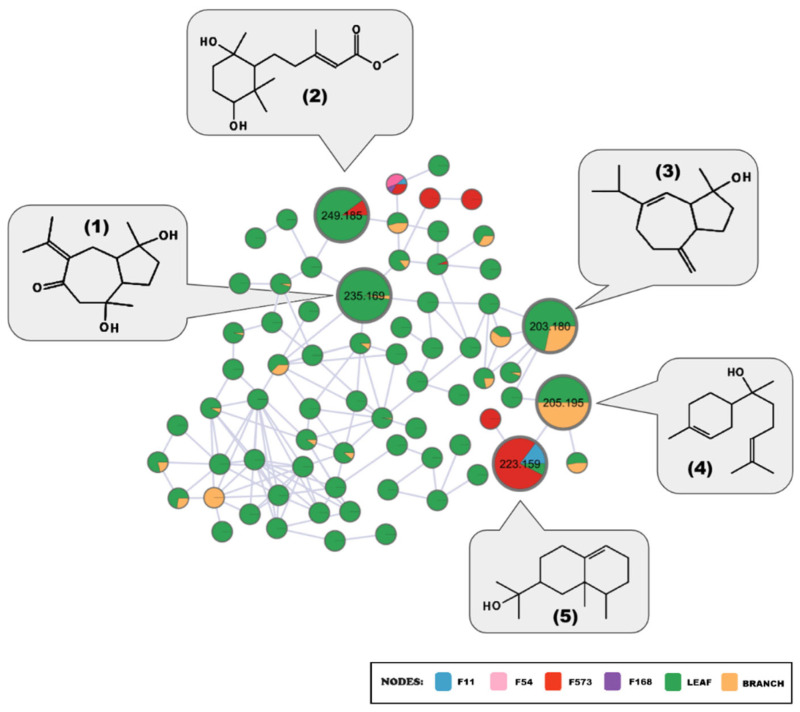
Compounds putatively identified as belonging to the sesquiterpene class: F573 (*P. chermesinum*), F11 (*P. sumatraense*), F54 (*P. miczynskii*), and F168 (*P. osmophilum*). (1) zedoarondiol; (2) canangalia H; (3) alismol; (4) alpha-bisabolol; (5) valerianol.

Two metabolites detected in both biological matrices were putatively identified as the sesquiterpenes canangalia H and valerianol (Figure 5). Within the same cluster, putatively identified by the GNPS library, alpha-bisabolol, detected in the leaf extract and branch extract, is connected to node *m*/*z* 223.159, detected in the leaf extract and fungal extracts F11, and in a higher percentage in F573. This indicates a strong structural relationship between them. Thus, using the propagation identification method, it is suggested that node *m*/*z* 223.159 can be assigned to the metabolite valerianol, which has a corresponding mass and was indicated with a high score by in silico fragmentation using MetFrag, Web beta, 2.6.12. The other two sesquiterpenes were detected in the plant extracts and putatively identified as zedoarondiol and alismol.

### 2.3. Metabolomic Profile of A. jahnii Extracts and Fungal Extracts

The study of the chemical profile of *A. jahnii* extracts allowed the putative identification of 41 compounds (Table S1). The compounds belong to different classes of metabolites, with alkaloids being predominant, in addition to terpenoids, flavonoids, and phenylpropanoids. Regarding the total number of unique and shared molecular characteristics, leaf extracts showed a greater number of molecular characteristics (816) compared to branch extracts (692). The greatest chemical diversity was obtained using ethyl acetate as the extraction solvent, resulting in 394 molecular characteristics detected in the leaf and 420 in the branch. For hexane and chloroform, the yields and the number of molecular characteristics were lower. In the leaves, the hexane extract showed a yield of 4.41% (272 molecular characteristics) and the chloroform extract, 1.98% (313 molecular characteristics). In the branch, the yields were 1.04% for the hexane extract (134 molecular characteristics) and 0.51% for the chloroform extract (238 molecular characteristics). Methanol showed the highest mass extraction yields, with 17.65% w/w for the leaves and 9.51% w/w for the branch, but showed the lowest chemical diversity, with 178 molecular characteristics for the leaf and 162 for the branch.

For fungal extracts, spectral analysis and Molecular Network approaches also proved to be useful tools for obtaining important information from this large and complex quantitative dataset obtained from fungal extracts. It was possible to identify 15 compounds (Table S1), such as the alkaloids viridicatine, viridicatol, cyclopeptine, cyclopenine, and cyclopenol, detected in extracts F573, F54, and F168. Cyclopenine was also detected in extract F11. These alkaloids belong to the same biosynthetic pathway, derived from anthranilic acid and phenylalanine, and are commonly isolated from fungal extracts of the genera *Penicillium* and *Aspergillus* [29,30].

### 2.4. Analysis of Antioxidant Activity

Table 1 presents the results obtained for the antioxidant activity of the tested extracts. The extracts obtained from the fungal strains F168 and F54 required the lowest concentrations to scavenge 50% of DPPH radicals (IC50), being 12.0 and 9.0 µg·mL^−1^, respectively. For the plant matrix, the ethyl acetate and methanol extracts from the leaf showed antioxidant activity of 12.0 and 11.0 µg·mL^−1^, the same result presented by the fungal extracts F54 and F168. With the exception of the extract from the F11 strain, the fungal extracts studied here showed better antioxidant activity compared to the results of the branch extracts.

## 3. Discussion

### 3.1. Metabolites Detected in Plant and Fungal Extracts and Analogs

The results of the comparative chemical profile of fungal and plant extracts showed the ability of endophytic fungi to produce the same metabolite as their host, not being restricted to the production of compounds specific to the plant organ from which the microorganism was isolated. It is important to highlight that this result can also be assigned to the similarity of the endophytic community in different parts of the plant, consistent with studies that demonstrate that an endophytic microorganism can be distributed in different plant parts through systemic colonization [31,32]. According to Ding et al., 2018 [12], endophytes can provide the accumulation of bioactive metabolites in the host plant. This information may, conversely, indicate that some of the metabolites obtained from the plant were actually produced by its endophytes. Regardless of the origin of the bioactive metabolites, the data obtained from this study confirm that fungal metabolomics exploration is advantageous and promising in the discovery of endophytic fungi that produce metabolites previously obtained only from the host plant.

Metabolomic analysis allowed the putative identification of four compounds detected in both matrices. Two of the metabolites were suggested by the GNPS library, with sinapic acid detected in the leaf extract and in the extract of the fungus F573, which is widely distributed in the plant kingdom and exhibits a wide range of biological activities, including antioxidant, antimicrobial, anti-inflammatory, anticancer, and anxiolytic [33]. Sinapic acid has already been identified in extracts of Brazilian *Annona* species that showed potent anticholinesterase and antiproliferative activities against tumor cells [34]. This is the first report of the production of this acid in an endophytic fungus and in the host plant, showing an equitable abundance of ions in both biological matrices, as observed in the pie chart. The second metabolite, canangalia H, detected in the fungal extract F573 and leaf extract, has already been isolated from the plant *Cananga latifolia*, also belonging to the Annonaceae family [35,36]. This metabolite is an analog of juvenile hormone III, a compound that, although uncommon in higher plants, is believed to be synthesized by plants as a defense mechanism against insects [35,37].

The identification propagation analysis allowed the dereplication of two more metabolites present in fungal and plant extracts. This analysis is widely used in MN dereplication [24,38,39]. It is based on the interpretation of mass differences, searching databases for compounds with common substructures, and in silico fragmentation performed on the MetFrag platform. MetFrag is a very useful tool when reference spectra are not available, contributing to the selection of more likely candidates, as well as increasing the reliability and number of putative identifications [40,41]. The two putatively identified metabolites were coumaryl acetate, detected in fungal extracts F54, F168, and F573, and in the branch extract. This metabolite has already been isolated from the rhizome of *Alpinia galanga*, which has phenylpropanoids as its main constituents and has shown significant phytotoxic and phytopathogenic activity [42]. The second metabolite, valerianol, present in the leaf extract and in fungal extracts F11 and F573, exhibits moderate inhibitory activity with cytotoxicity in Raji cells [43].

Other compounds analogous to those detected in both matrices have also been putatively identified, standing out for their significant biological properties. Tiliroside shows potential as an antioxidant and anti-inflammatory agent [44]. Paprazine exhibits an inhibitory effect on acetylcholinesterase [45] and feruloyltyramine may have an action against SARS-CoV-2 [46]. Both have already been detected in *Annona* species [47,48]. *N*-*trans*-Feruloyloctopamine appears as an inhibitor of proliferation and a stimulator of the apoptosis of Huh7 and Hep3B tumor cells [49].

The presence of compounds detected in both biological matrices, as well as analogous compounds, reinforces that despite successive cultivation in the laboratory, these fungi have the ability to biosynthesize metabolites present in their host plant independently of the plant’s unique environment, although the lack of host stimuli can decrease this biosynthetic capacity [12,13,14,15]. This study, therefore, presents an extremely useful and rapid strategy for exploring microorganisms capable of producing metabolites previously obtained exclusively from the host plant. The investigation of this capacity in most studies is directed and limited to the identification of one or a few specific metabolites present in the plant and produced by its fungus [7,50,51,52,53].

### 3.2. Chemical Profile of Plant and Fungal Extracts

The study of the chemical profile allowed for the putative identification of compounds unique to plant extracts. Some of these compounds have been previously reported, occurring in the Annonaceae family and showing promising biological activity. Notable examples include feruloylthyramine with potent cytotoxic activity against the P-338 and HL-60 cell strains [54]; the aporphine alkaloid asimilobine with significant antimicrobial and antioxidant activities [55]; and naringenin, a flavonoid with anti-glycemic properties that gives protection against diabetes [56]. The analysis of plant extracts revealed significant differences both between the plant parts evaluated and the solvents used in extraction. The results indicate a greater chemical complexity associated with the leaves, regardless of the solvent used. Extracts obtained from the leaves are more promising for obtaining a greater variety of metabolites, since they presented a greater number of molecular characteristics. Considering the chemical diversity, the extracts obtained with ethyl acetate showed the most significant results, suggesting that this solvent favors the extraction of a greater variety of metabolites, even with lower mass yields than those observed for methanol.

The study of leaf and branch extracts generates information about the chemical profile common to both, an interesting investigative path regarding the dynamics of the production and distribution of these substances in plant tissues.

The analysis of the chemical profile of fungal extracts revealed a large and complex amount of data and resulted in the putative identification of compounds that possess important biological activities, such as the inhibition of cancer cell metastasis, an activity exhibited by viridicatin and viridicatol [57]. Cyclopenine is indicated as a useful anti-inflammatory agent for neurodegenerative diseases [58].

The results demonstrate the need to expand the metabolomic exploration of the Amazonian microbiota in order to assist future research related to the biotechnological potential of these microorganisms. The scarcity of chemical studies on the species *A. jahnii* also points to the need for studies similar to those developed in this research to expand the chemical knowledge of this species, which has shown promise in obtaining bioactive compounds. The metabolomic study provided the detection of a broad and varied spectrum of compounds, providing unprecedented information about *A. jahnii* and its endophytic fungi, which were previously unexplored.

### 3.3. Antioxidant Activity

Preliminary analysis of the antioxidant activity indicated that the fungal strains studied have extracts with superior antioxidant potential compared to other *Penicillium* species tested in other studies, such as *P. fumiculosum* and *P. chrysogenum*, which showed antioxidant activity at 1000 µg·mL^−1^ [59]. *P. thomii*, with an antioxidant activity of 470 µg·mL^−1^, even when supplemented with selenium, showed higher values for this property [60]. The fungal strains also showed significantly more promising antioxidant activity than the extracts obtained from the branch of *A. jahnii* and equivalent to the results obtained for the leaf extracts, demonstrating the high potential of these endophytic fungi for this biological activity compared to the host plant. This result reinforces the viability of using endophytic fungi as an alternative resource to plants, potentially replacing them in the obtention of bioactive metabolites.

## 4. Materials and Methods

### 4.1. Plant Material

The plant *A. jhanii* Saff. was collected in its natural habitat, in Roraima (N 03°06′43.2″ and W 060°52′04.1″), Brazil. The leaves and branches were dried in an oven at 40 °C until constant mass and pulverized in a knife mill. A 150 g sample of the pulverized material from each part was subjected to sequential extraction using a Soxhlet apparatus with the following bidistilled solvents: hexane (98%, Synth, Diadema, SP, Brazil), chloroform (98%, Synth, Diadema, SP, Brazil), ethyl acetate (98%, Synth, Diadema, SP, Brazil), and methanol (98%, Synth, Diadema, SP, Brazil) for 12 h. After extraction, the solutions were dried with Na_2_SO_4_ (98%, Synth, Diadema, SP, Brazil).

### 4.2. Endophytic Fungi

A sample of each endophytic fungus was sent for amplification and sequencing of the internal spacer region transcribed from nuclear ribosomal tissue (ITS). The four fungal strains isolated from *A. jhanii* were identified as belonging to the genus *Penicillium*, to which the following identification codes were assigned: F11, F54, F168, and F573. In the phylogenetic analysis, the ITS1–5.8S rDNA–ITS2 sequence grouped the strains close to the clades of *Penicillium sumatraense*, *Penicillium miczynskii*, *Penicillium osmophilum*, and *Penicillium chermesinum*, respectively.

### 4.3. Cultivation Conditions and Preparation of Fungal Extracts

The culture media and fungal extracts were prepared according to Xavier et al., 2022 [61]. Fungi F573 (*P. chermesinum*) and F11 (*P. sumatraense*) were cultivated in liquid oat medium, and fungus F54 (*P. miczynskii*) in a medium containing starch. The F168 (*P. osmophilum*) was cultivated in potato dextrose prepared according to Souza et al., 2004 [62]. After the cultivation period, the mycelium was separated from the liquid medium by vacuum filtration. The liquid media were extracted with bidistilled ethyl acetate (Synth, Diadema, SP, Brazil), and the organic fractions were dried using a rotary evaporator.

### 4.4. 2,2-Diphenyl-1-picryl-hydrazyl (DPPH) Radical-Scavenging Activity Analysis

The antioxidant activity of all fungal and plant extracts was measured using a solution of the reagent 1,1-diphenyl-2-picrylhydrazyl (DPPH, purity > 90%, Aldrich, St. Louis, MO, USA). The method was developed according to that described in Pontis et al., 2014 [63]. Different sized aliquots of the solutions for all extracts (1 mg mL^−1^) were withdrawn, followed by the addition of 1.5 mL DPPH (1 mmol L^−1^). A solution consisting of 1.5 mL of DPPH added with methanol was also prepared and used as a control. The blank was formed by the same aliquots of samples and methanol. Determinations were performed using a UV-Vis spectrophotometer (Shimadzu UV-mini model 1240, Tokyo, Japan).

All assays were performed in triplicate, calculating the values of the antioxidant concentration required to scavenge 50% of DPPH radicals (IC50) from linear regression of the plots (Origin 6.0 software) and the following formula:

AA% = 100 − {[(Abssample − Absblank) × 100]/Abscontrol}

### 4.5. UHPLC-ESI-Q-TOF-MS/MS and Molecular Networking

MS2 spectra of fungal and plant extracts were obtained from analyses performed on an ultra-high performance liquid chromatograph (UHPLC) (Shimadzu, Nexera X2, Tokyo, Japan), with an ACQUITY UPLC CSH C18 column (130 Å, 2.1 × 100 mm, 1.7 μm), coupled to a high-resolution mass spectrometer (HRMS) Impact II (Bruker Daltonics Corporation, Bremen, Germany) with Q-TOF geometry and an electrospray ionization source. The analysis conditions are described in Table 2. Data were acquired using Hystar Application software version 3.2 and Otof Control version 3.4 (Bruker Daltonics Corporation, Bremen, Germany).

For the development of the Classical Molecular Networking (MN), mass spectrometry data in mzXML format were transferred to the Global Natural Products Social Molecular Networking (GNPS) server [25]. The mzXML files of all branch extracts were grouped together, and the mzXML files of the leaf extracts were also grouped together. The MN of the fungal and plant extracts was then generated using the online workflow (https://ccms-ucsd.github.io/GNPSDocumentation/) (accessed on 25 november 2025) [25]. The precursor ion mass tolerance was set to 0.02 Da and the MS/MS fragment ion tolerance to 0.02 Da. A cosine score above 0.7 and more than 4 corresponding peaks were applied. A detailed description of the parameters used in MN can be accessed via the following link: https://gnps.ucsd.edu/ProteoSAFe/result.jsp?task=faef3d81c9684797ab8552a4b443187e&view=written_description, (accessed on 25 november 2025).

The annotation of metabolites followed the workflow described in Xavier et al., 2022 [61]. Spectra from GNPS spectral libraries were filtered in the same way as the input data. Only compounds of interest (annotated or analogs) were subjected to computer-assisted in silico fragmentation, using the MetFrag (2.6.12) web tool.

## 5. Conclusions

The study of endophytic fungi for obtaining bioactive compounds is a promising alternative to meet the increasing demand for these resources from a host plant, reducing the impacts of plant exploitation and potentially increasing their industrial-scale production through fermentation. However, research aimed at efficiently and rapidly identifying endophytes capable of biosynthesizing the same or similar active substances in relation to those produced by the host plant remains a challenge. This study reveals the use of UHPLC/ESI-MS/MS analyses combined with Molecular Networks and annotation techniques as a simple, fast, and powerful strategy, allowing for a preliminary and efficient evaluation of crude extracts of endophytic fungi and their host plants, which represent a complex mixture, for the detection and dereplication of plant/fungus compounds identical and analogous to the metabolites present in the plant.

The obtained results provided unprecedented information regarding the chemical profile of *A. jahnii* and its endophytic fungi. The large quantity of compounds from different chemical groups, present in both biological matrices, demonstrates the potential of microorganisms in the production of bioactive metabolites and plant derivatives. The findings presented in our study contribute to the expanding knowledge of the metabolome of endophytic fungi from the Amazonian biome, which is still scarce. This research provides new perspectives for the exploration of these fungi, which should be expanded and followed by further studies, aided by the strategy presented here.

## Figures and Tables

**Table 1:** Results of antioxidant activity analyses by DPPH.

Extracts	IC_50_ (µg·mL^−1^)
F11 (*P. sumatraense*)	77.0 ± 4.9 × 10^−3^
F54 (*P. miczynskii*)	11.0 ± 1.5 × 10^−3^
F168 (*P. osmophilum*)	11.0 ± 1.0 × 10^−3^
F573 (*P. chermesinum*)	18.0 ± 5.8 × 10^−4^
Methanol leaf extract	11.0 ± 5.8 × 10^−4^
Ethyl acetate leaf extract	12.0 ± 1.5 × 10^−3^
Methanol branch extract	22.0 ± 1.0 × 10^−3^
Ethyl acetate branch extract	46.0 ± 6.0 × 10^−4^

**Table 2:** Description of the analytical conditions applied in UHPLC for the obtention of mass spectrometry data.

Parameters	Conditions
Flow rate	0.200 mL
Mobile phase	Water (Mili Q) with 0.1% formic acid (98–100% for LC–MS LiChropour, Supelco), *v*/*v* (solvent A) a and acetonitrile (hypergrade for LC–MS, LiChrosorv) with 0.1% formic acid (solvent B)
Elution flow	(I) 2% B 0–1 min; (II) 30% B 1–3 min; (III) 80% B 3–20 min; (VI) 98% B 20–32 min; (V) 2% B 32–38 min
Column temperature	40 °C
Calibrated using a solution	Sodium formiate (10 mmol/L; isopropanol and water 1:1 *v*/*v*) containing 50 μL of formic acid
Ionization source	Positive mode and adjusted to 4500 V, with an end plate offset potential of 500 V
Drying gas	8 L·min^−1^, 180 ° C and nebulizing gas pressure of 4 bar
Range from m/z	50 to 1200 with an acquisition rate of 5 Hz
Auto MS/MS	The five most intense ions

## Data Availability

The original contributions presented in this study are included in the article/Supplementary Materials. Further inquiries can be directed to the corresponding author.

## Supplementary Materials

The following supporting information can be downloaded at: https://www.mdpi.com/article/10.3390/plants15030501/s1.
