# Supplementary material for: Metabolomic Profiling of Endophytic Fungi and the Host Plant Annona jahnii Saff. Reveals Shared and Analogous Compounds
Source: Plants (Basel). 2026 Feb 5;15(3):501. doi: 10.3390/plants15030501 (PMC12899986; doi:10.3390/plants15030501)
Supplement: Supplementary file 1 [file plants-15-00501-s001.zip › plants-4103401-supplementary.pdf]

Supplementary Materials

Figure S1: Mass spectra of the vanillic acid *m/z* 169.0968 [M+H]+ detected in the fungal extracts.

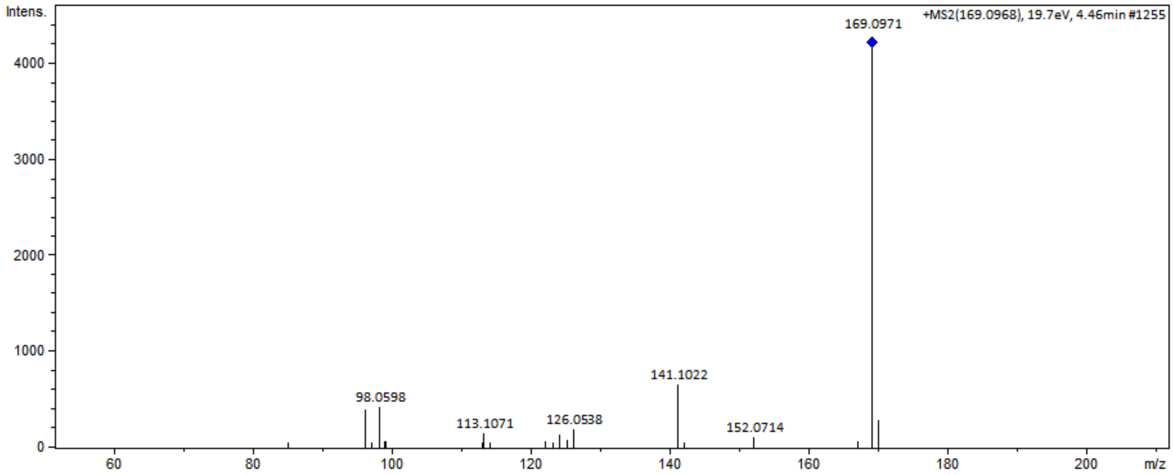

Figure S2: Mass spectra of the gamma-decalactone *m/z* 171.1374 [M+H]+ detected in the fungal extracts.

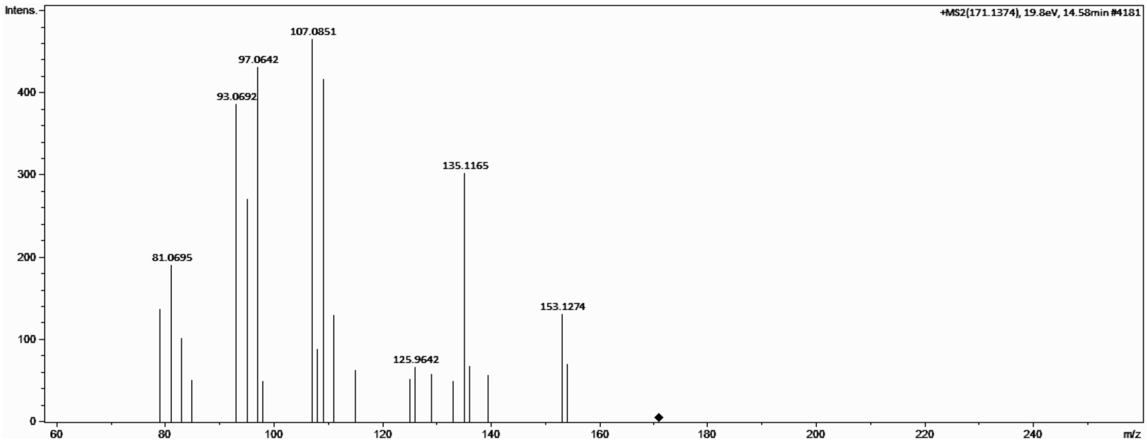

Figure S3: Mass spectra of the 3-Hydroxy-4-methoxycinnamic acid *m/z* 177.0543 [M+H]+ detected in the leaf and branch extracts.

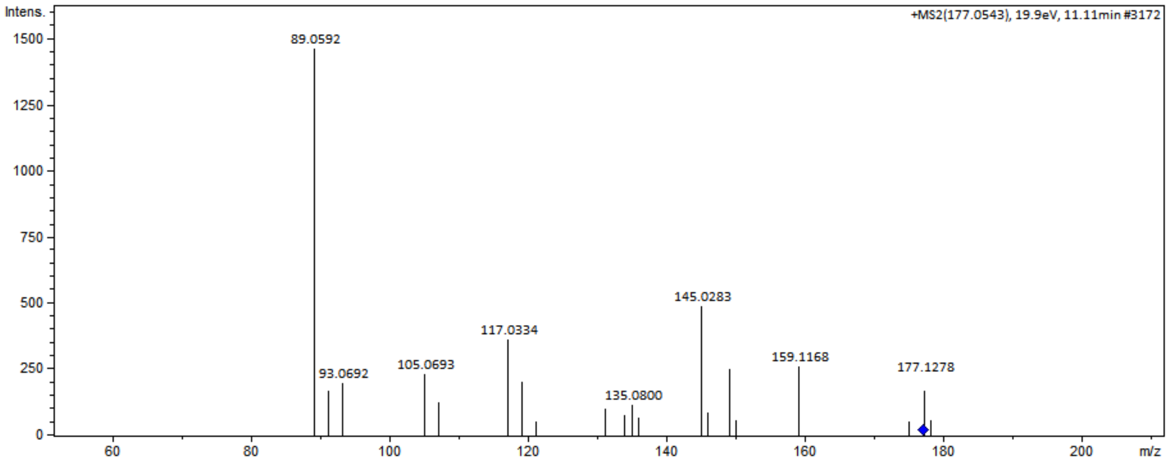

Figure S4: Mass spectra of the p-Acetaminobenzoic acid *m/z* 180.0649 [M+H]+ detected in the fungal extracts.

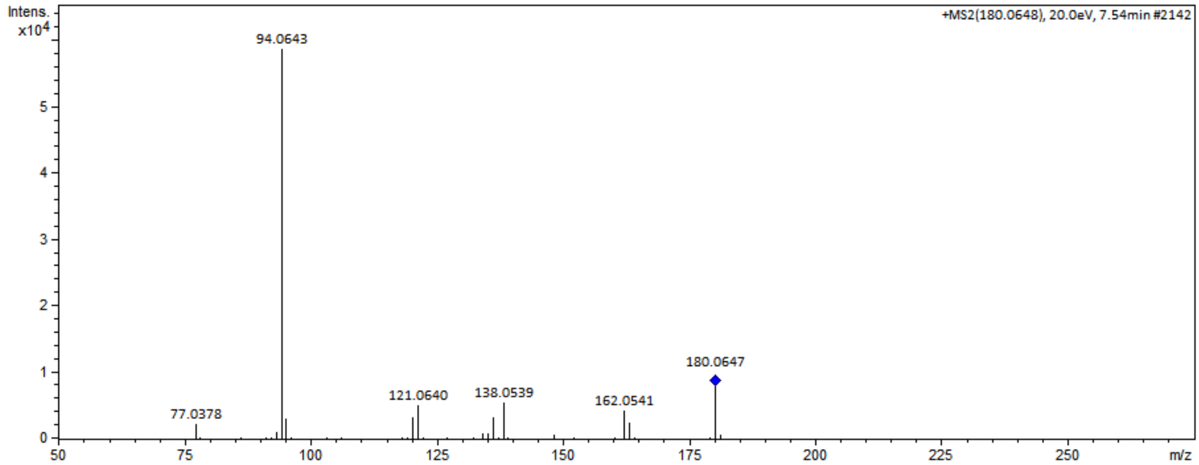

Figure S5:Mass spectra of the coumaryl acetate *m/z* 193.0701 [M+H]+ detected in the fungal extract and plant extracts.

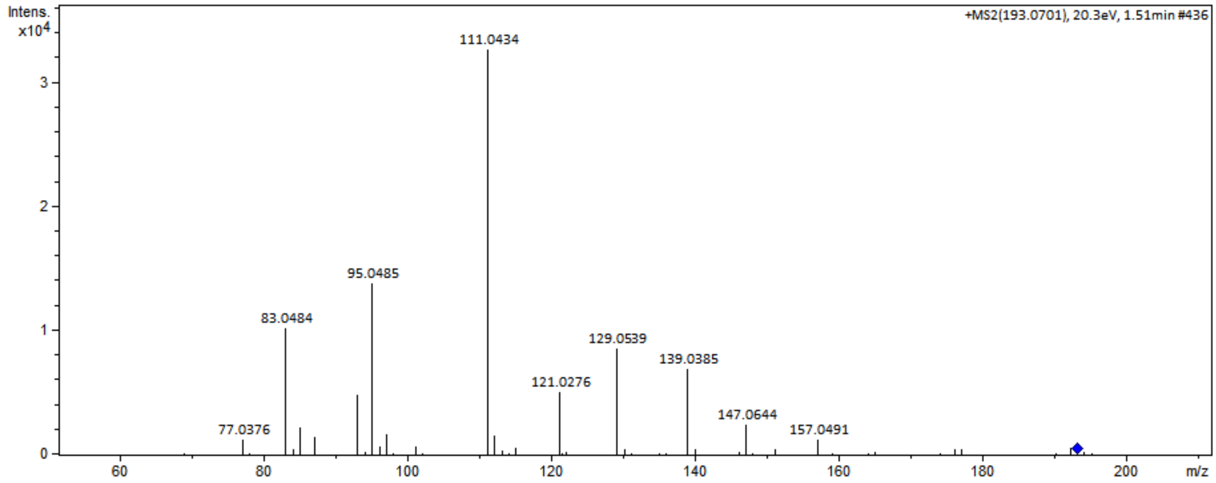

Figure S6: Mass spectra of the loliolide m/z 197.1168 [M+H]+ detected in the branch extract.

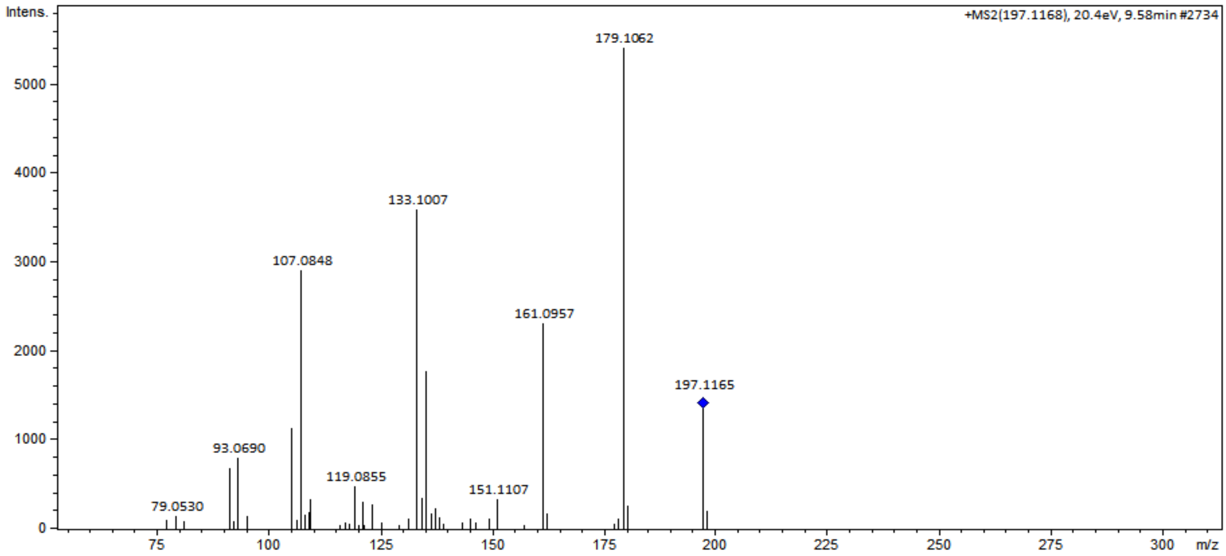

Figure S7: Mass spectra of the alismol m/z 203.1790 [M-H2O+H]+ detected in the f extract.

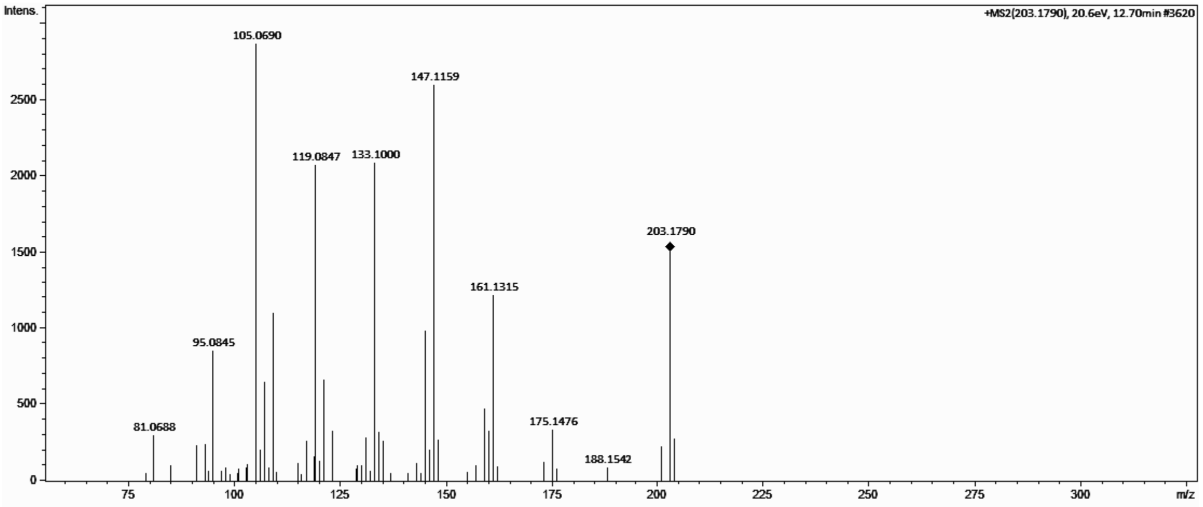

Figure S8: Mass spectra of the a-bisabol m/z 205.0813 [M+H]+ detected in the fungal extract and plant extracts.

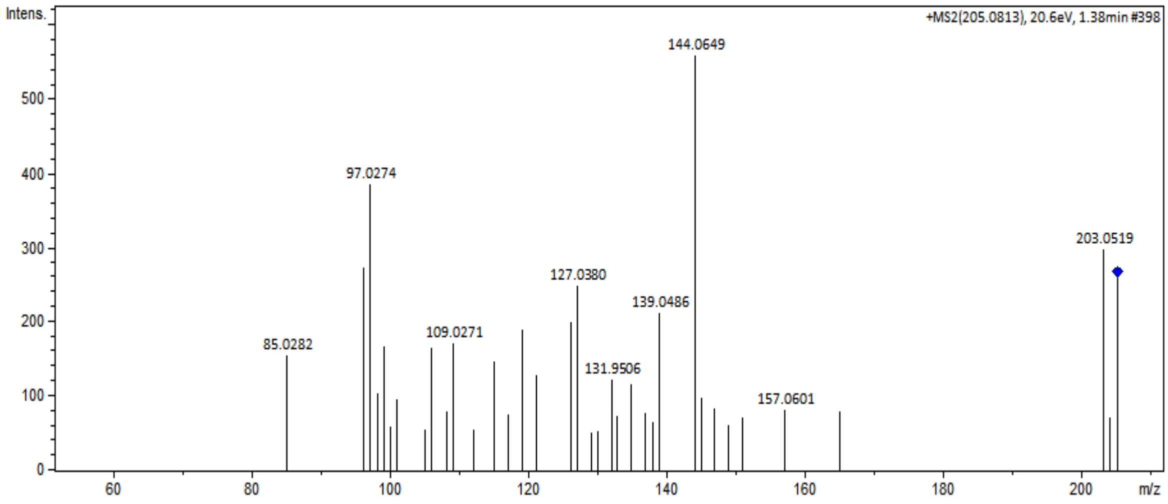

Figure S9: Mass spectra of the Jasmonic Acid m/z 211.1324 [M+H]+ detected in the plant extracts.

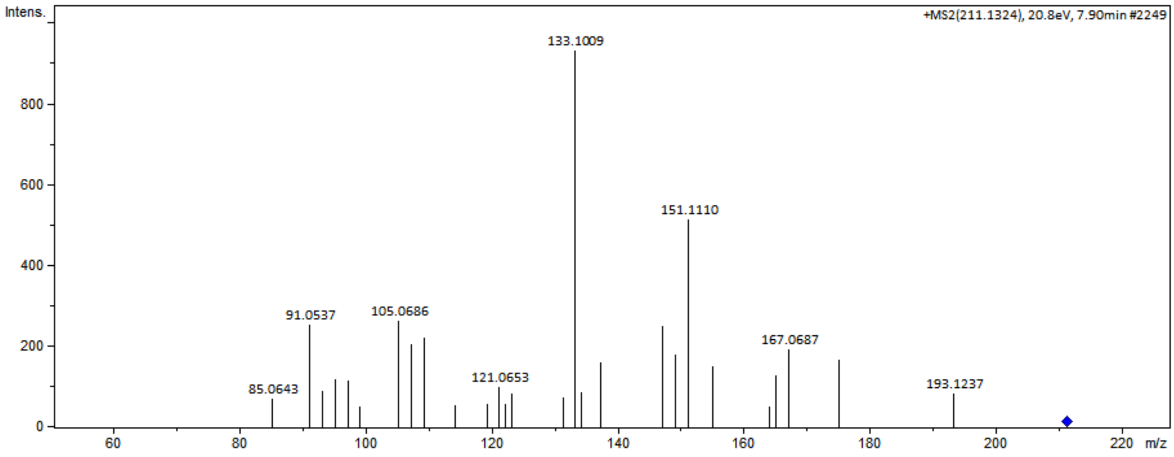

Figure S10: Mass spectra of the valerianol m/z 223.1680 [M+H]+ detected in the fungal extract and plant extracts.

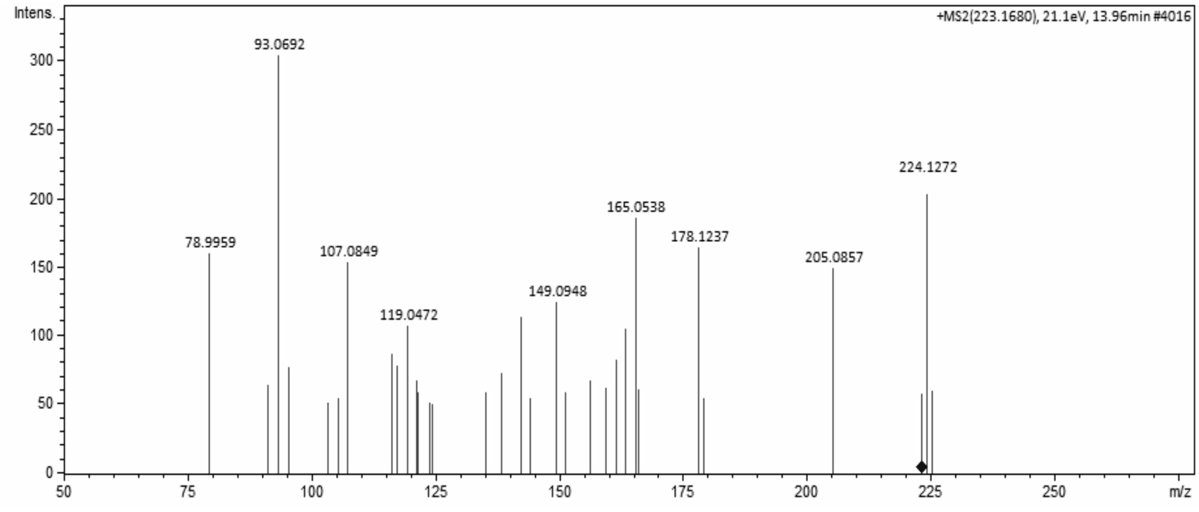

Figure S11: Mass spectra of the Sinapic acid *m/z* 225.0750 [M+H]+ detected in the fungal extract and plant extracts.

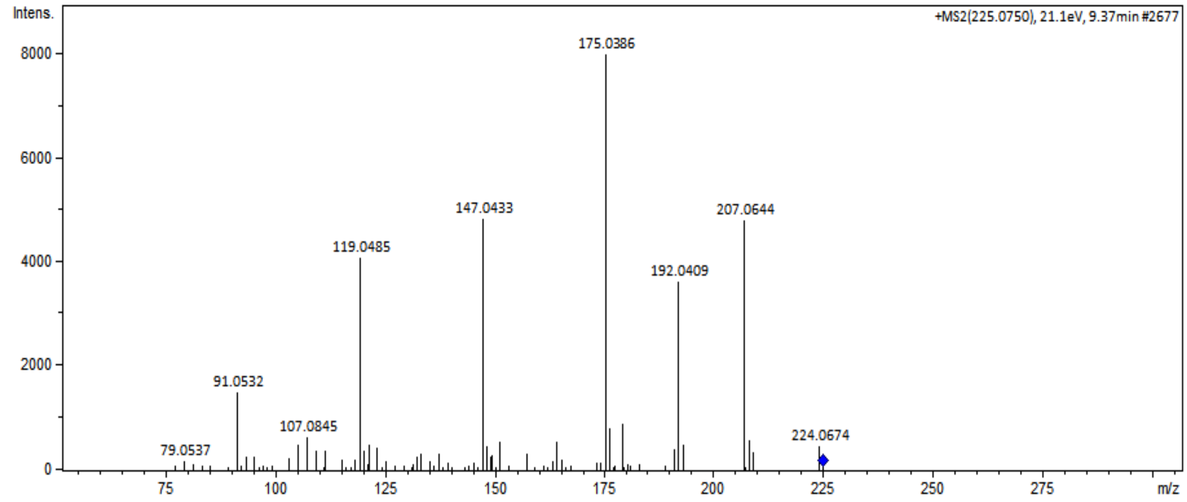

Figure S12: Mass spectra of the zedoarondiol*m/z* 235.1690 [M+H]+ detected in the leaf and branch extract.

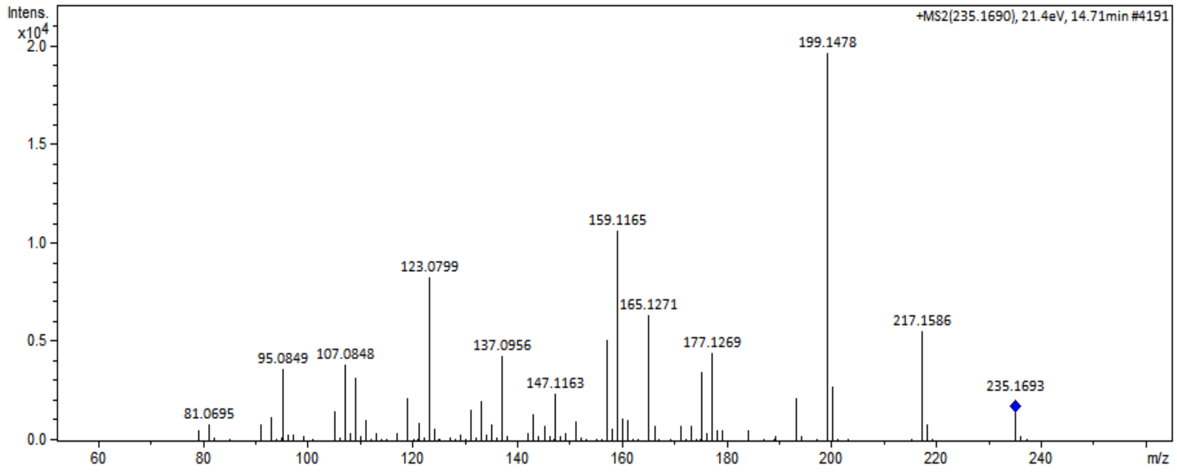

Figure S13: Mass spectra of the viridicatin *m/z* 238.0869 [M+H]+ detected in the fungal extract.

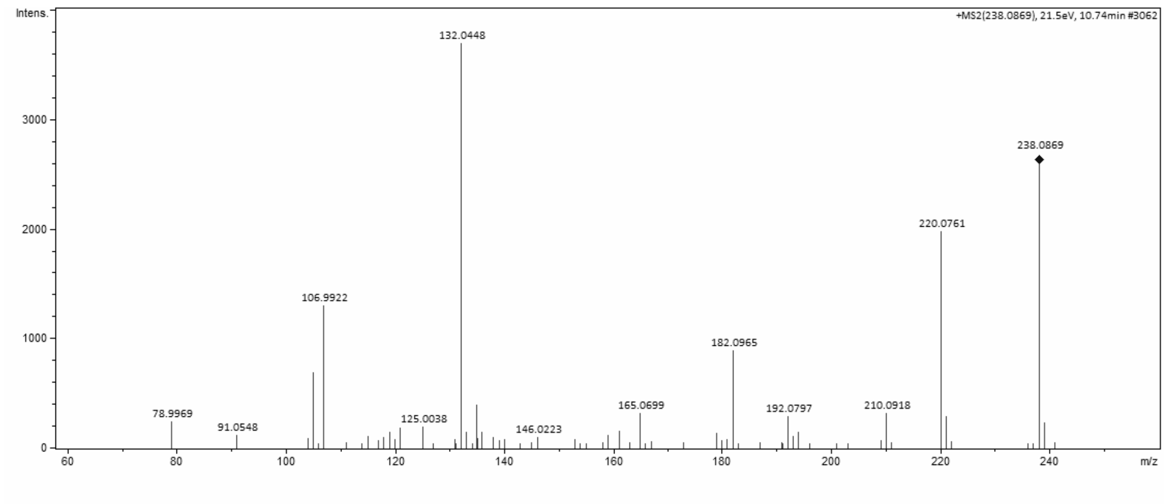

Figure S14: Mass spectra of the canangalia H *m/z* 249.1843 $[M-2H_2O+H]^+$ detected in the fungal extract and plant extracts.

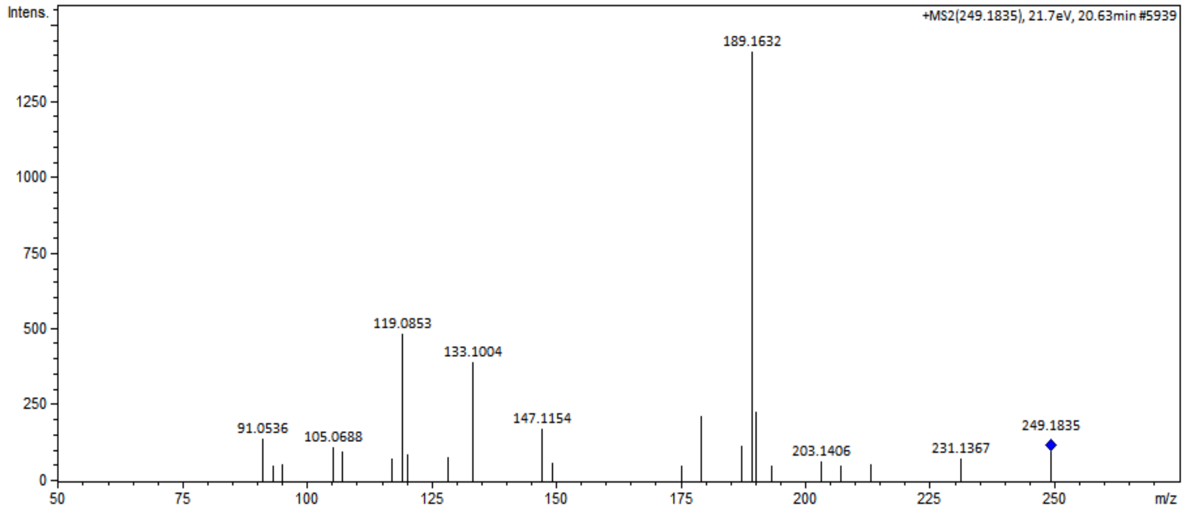

Figure S15: Mass spectra of the viridicatol *m/z* 254.0815 $[M+H]^+$ detected in the fungal extract.

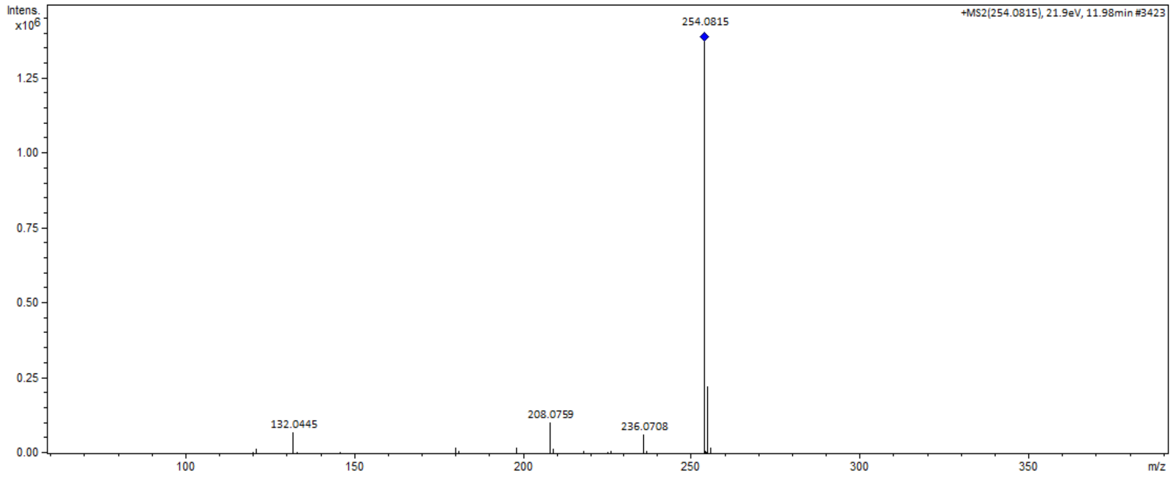

Figure S16: Mass spectra of the 4-phenyl-3,4-dihydroquinolin-2(1H)-one *m/z* 256.0971 $[M+H]^+$ detected in the fungal extract.

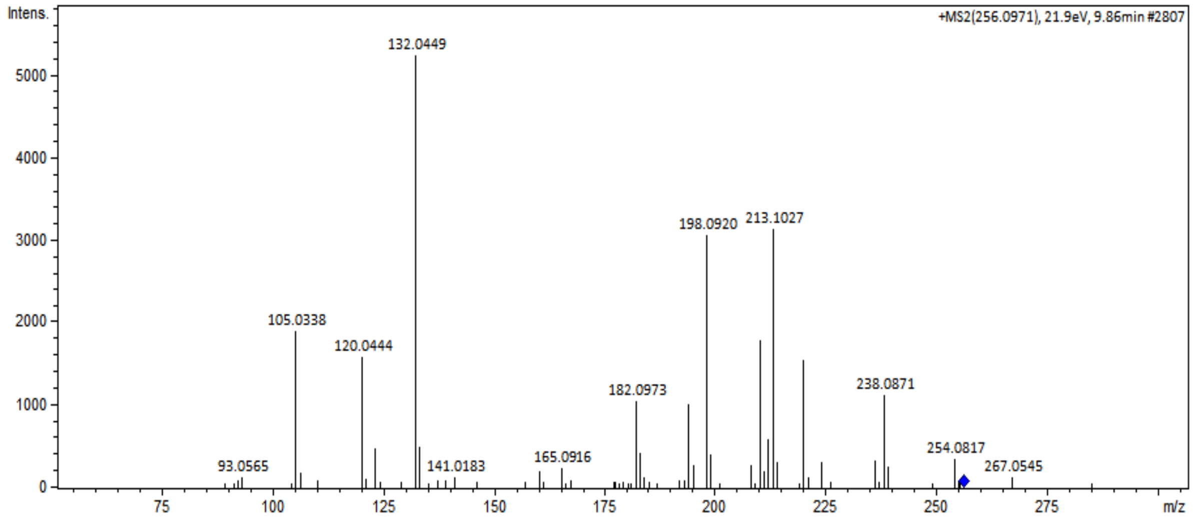

Figure S17: Mass spectra of the asimilobine *m/z* 268.1324 [M+H]⁺ detected in the leaf and branch extracts.

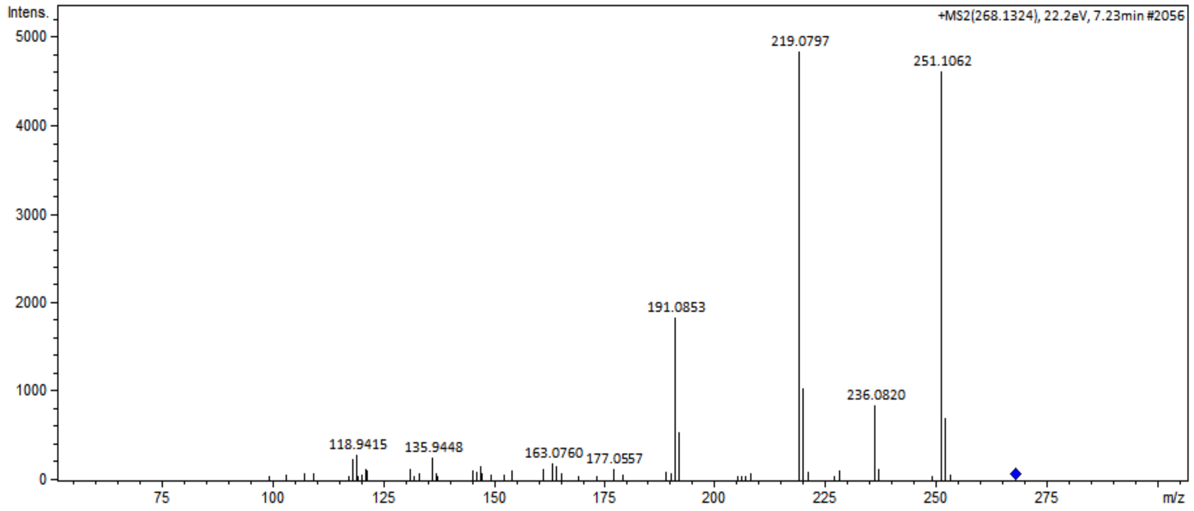

Figure S18: Mass spectra of the naringenin *m/z* 273.0753 [M+H]⁺ detected in the leaf and branch extracts
.

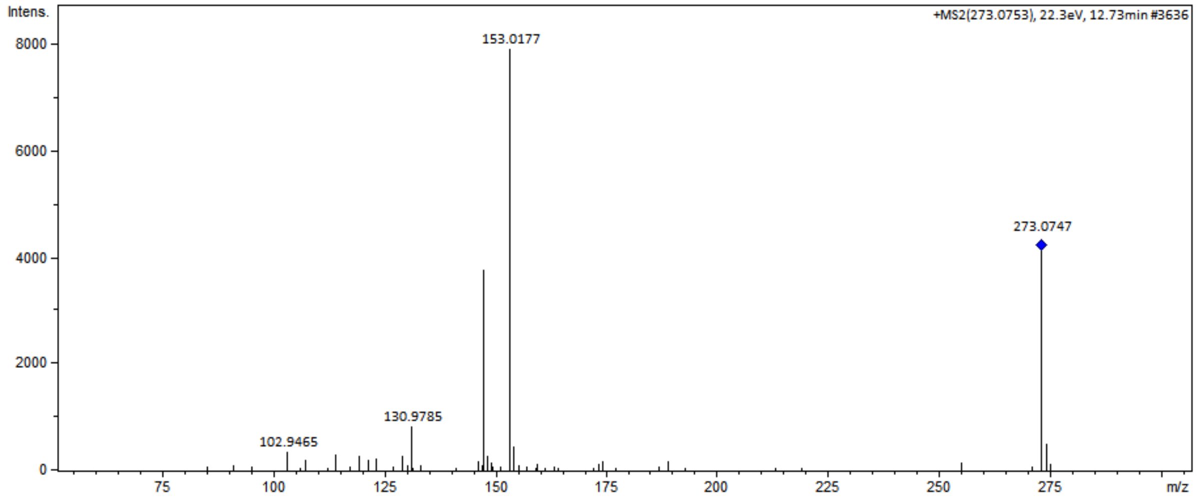

Figure S19: Mass spectra of the cyclopeptin *m/z* 281.1285 [M+H]⁺ detected in the fungal extracts.

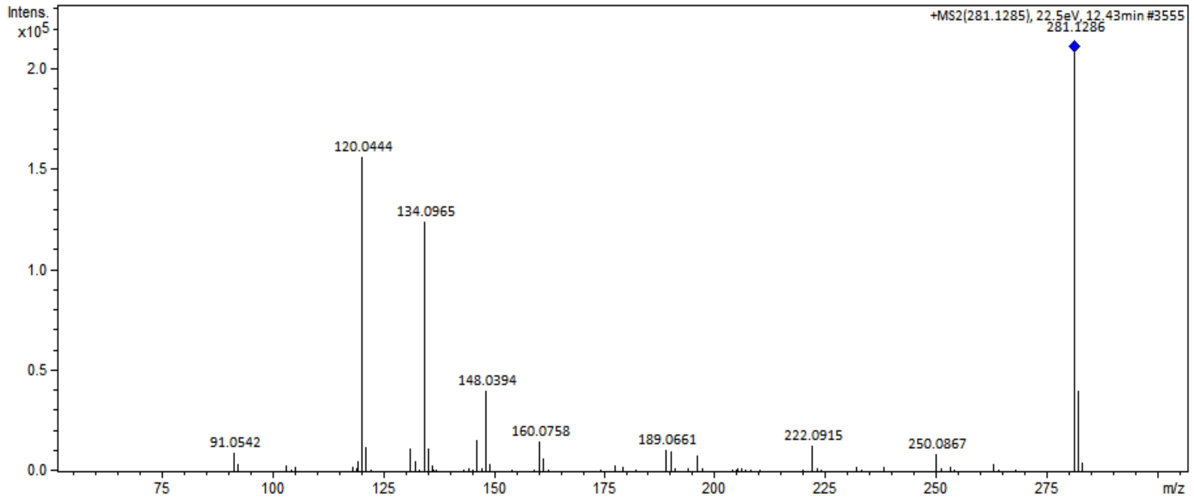

Figure S20: Mass spectra of the paprazine *m/z* 284.1280 [M+H]+ detected in the leaf and branch extracts.

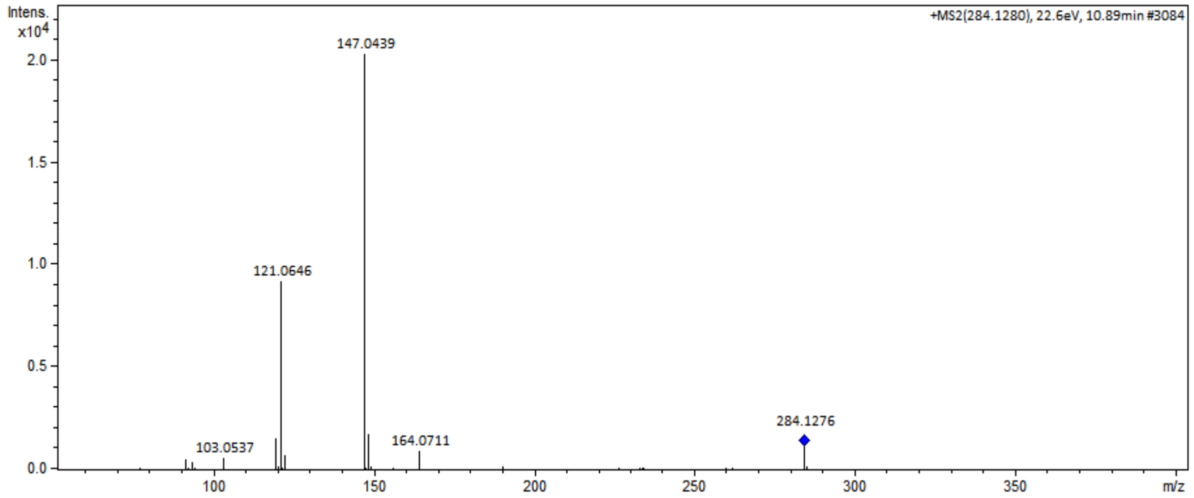

Figure S21: Mass spectra of the Coclaurin *m/z* 286.1434 [M+H]+ detected in the leaf and branch extracts.

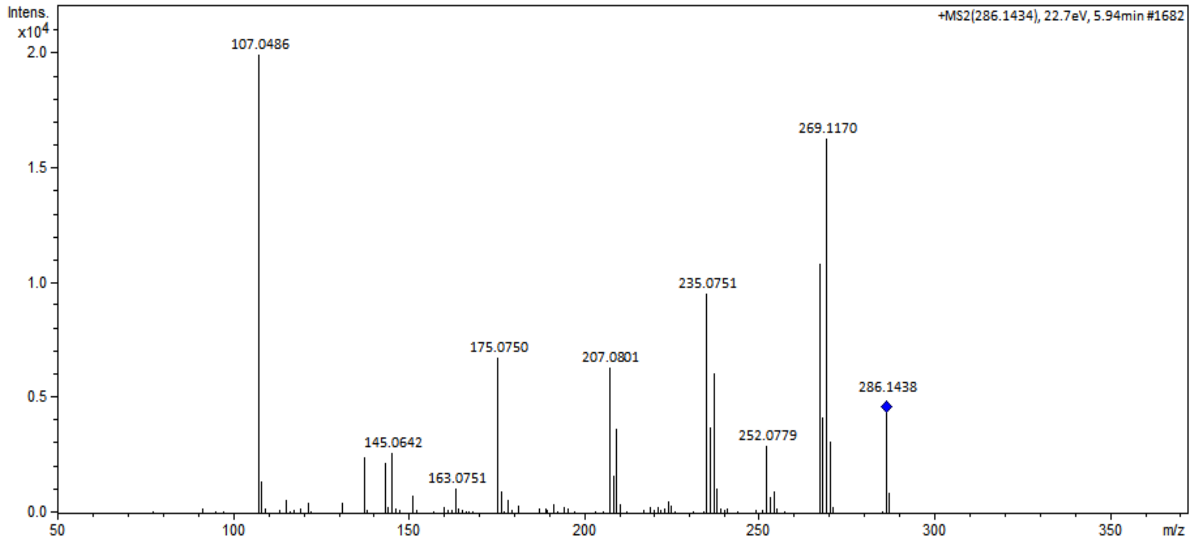

Figure S22: Mass spectra of the eriodictiol *m/z* 289.0705 [M+H]+ detected in the leaf and branch extracts.

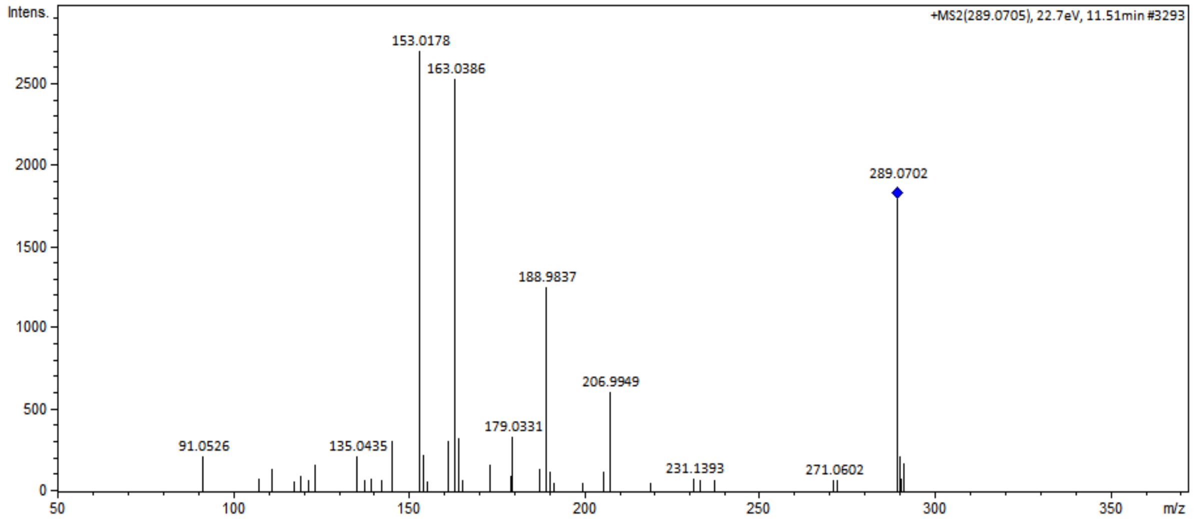

Figure S23: Mass spectra of the Epicatechin *m/z* 291.0860 [M+H]+ detected in the leaf and branch extracts

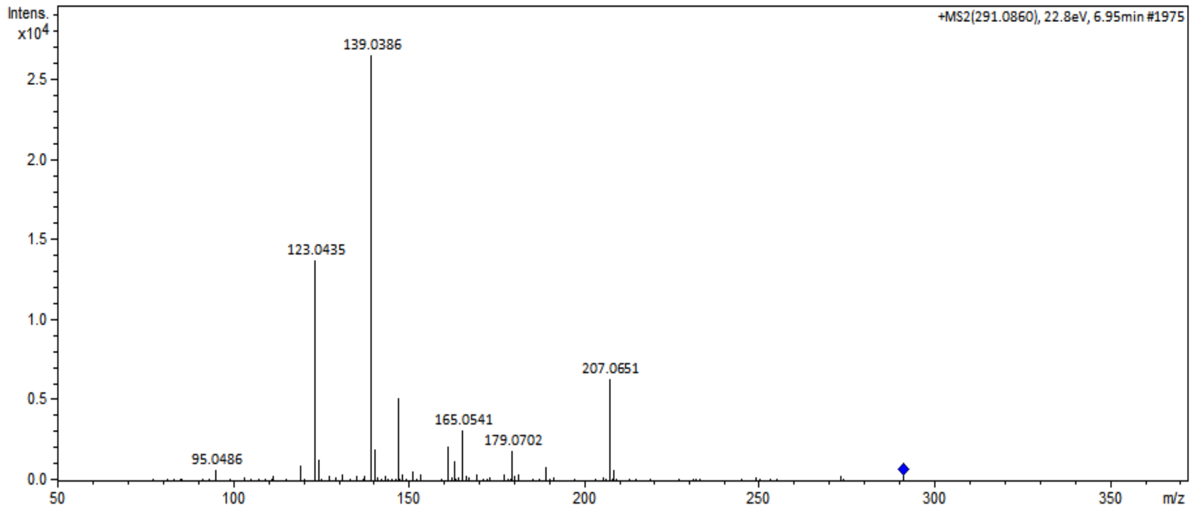

Figure S24: Mass spectra of the dehydrocurvularine *m/z* 291.1213 [M+H]+ detected in the fungal extracts.

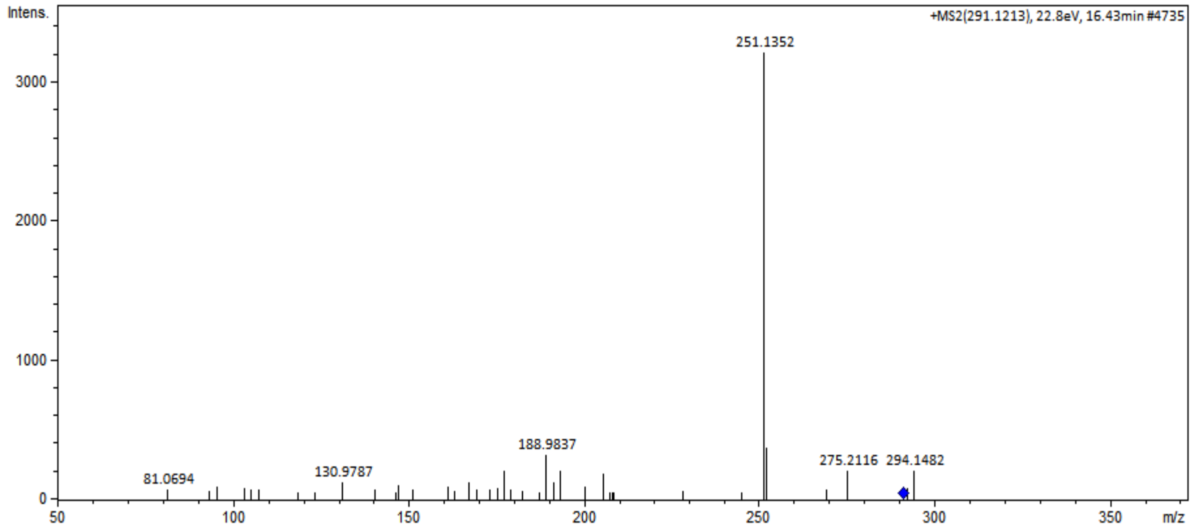

Figure S25: Mass spectra of the curvularin *m/z* 293.1367 [M+H]+ detected in the fungal extracts.

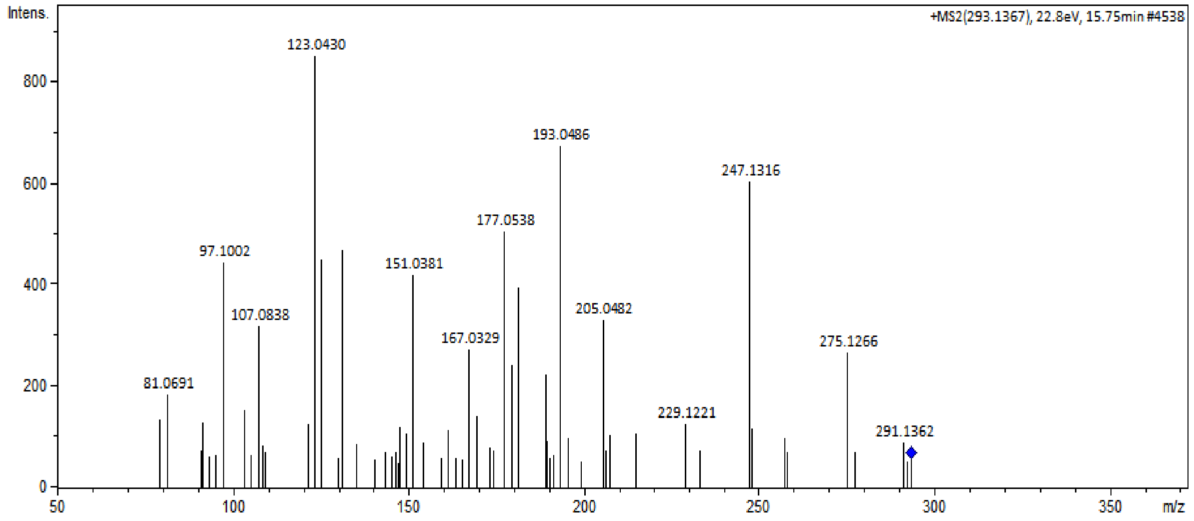

Figure S26: Mass spectra of the cyclopenin *m/z* 295.1080 [M+H]$^+$ detected in the fungal extracts.

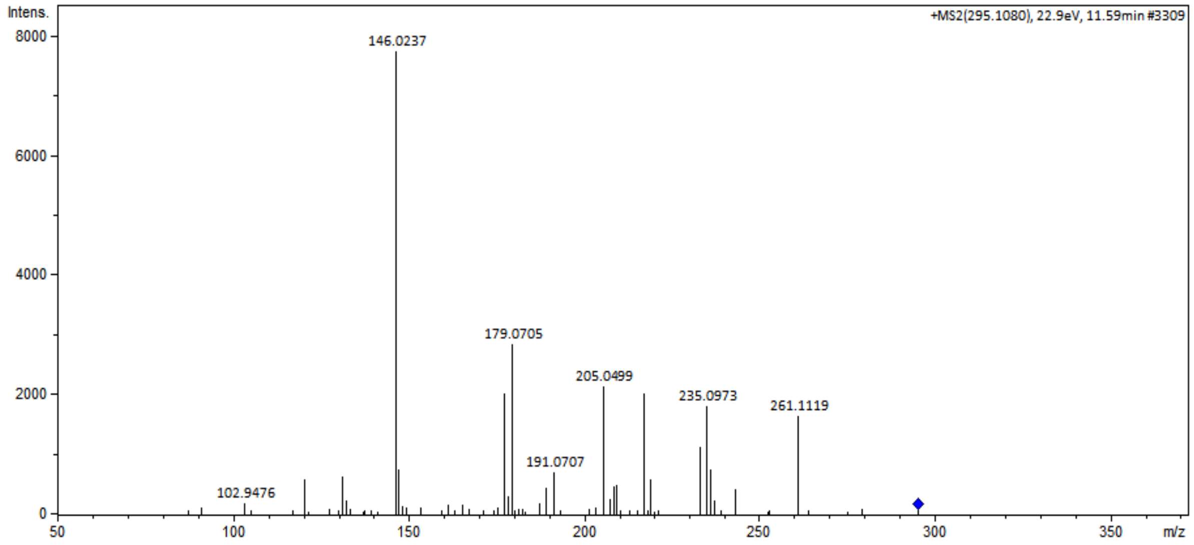

Figure S27: Mass spectra of the Stepharine *m/z* 298.1435 [M+H]$^+$ detected in the leaf and branch extracts.

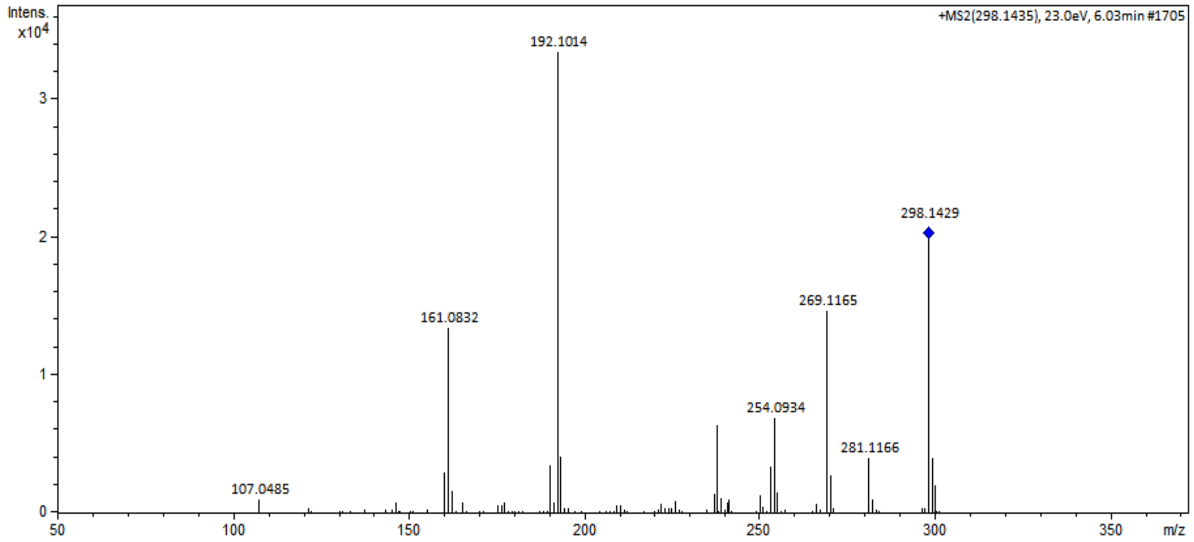

Figure S28: Mass spectra of the *N*-methylcoclaurine *m/z* 300.1594 [M+H]$^+$ detected in the leaf and branch extracts.

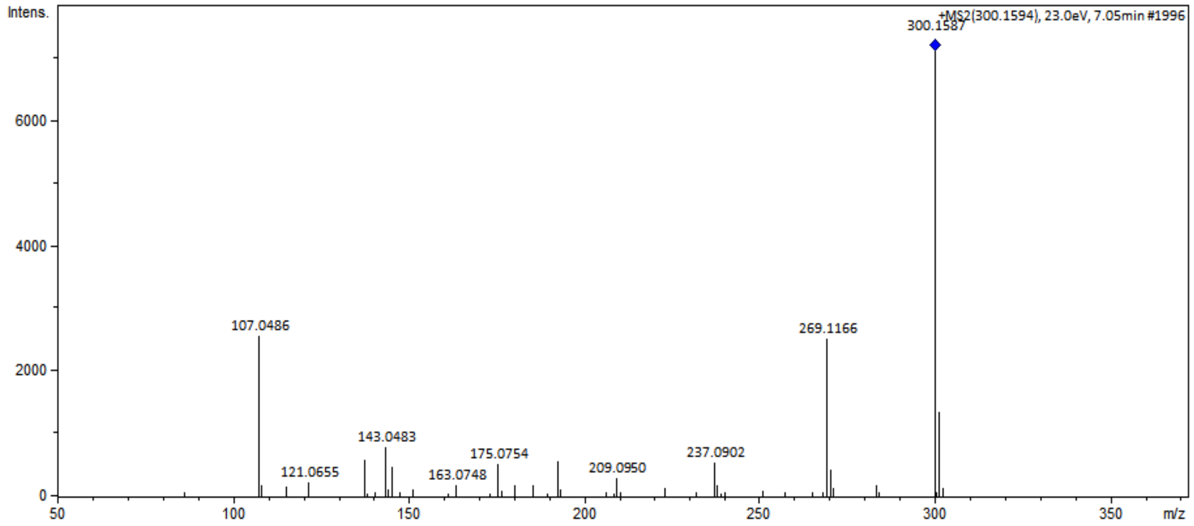

Figure S29: Mass spectra of the 5,7,3',4',5'-pentahydroxyflavanone *m/z* 305.0652 [M+H]⁺ detected in the leaf and branch extracts.

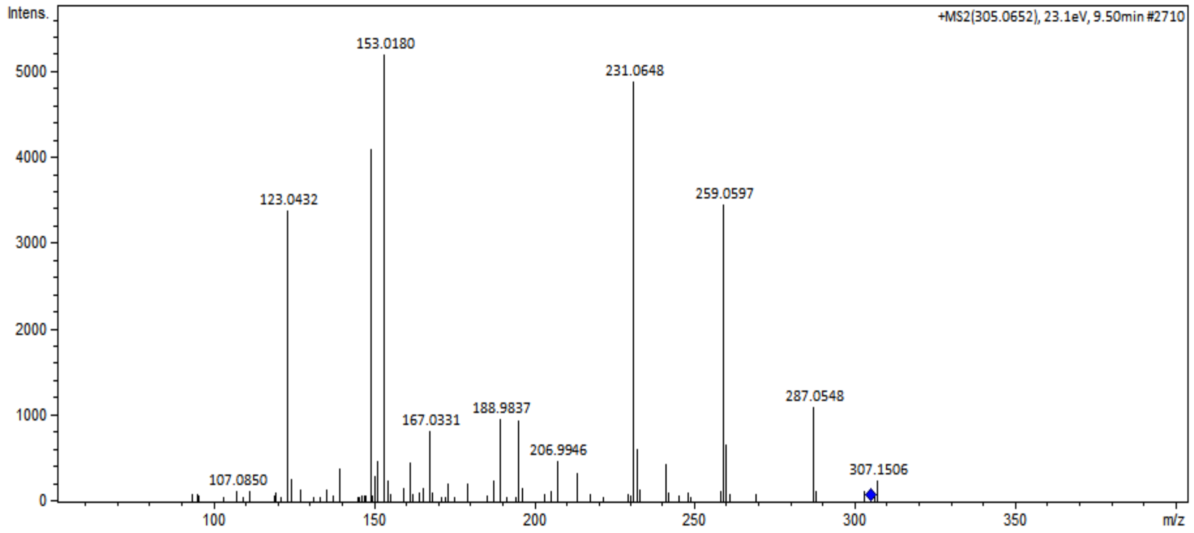

Figure S30: Mass spectra of the cyclopenol *m/z* 311.1032 [M+H]⁺ detected in the leaf and branch extracts.

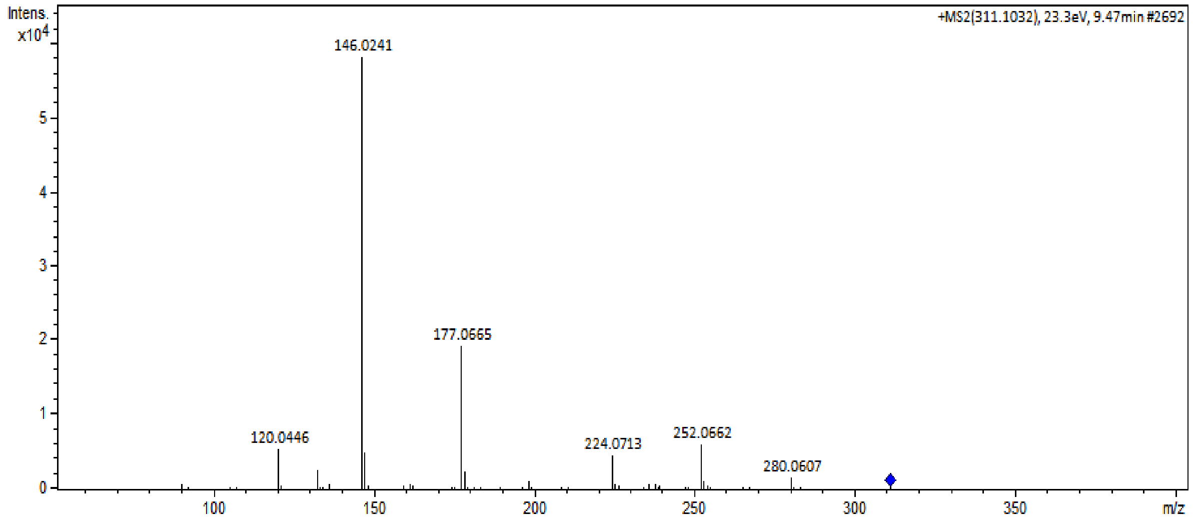

Figure S31: Mass spectra of the norboldine *m/z* 314.1382 [M+H]⁺ detected in the leaf and branch extracts.

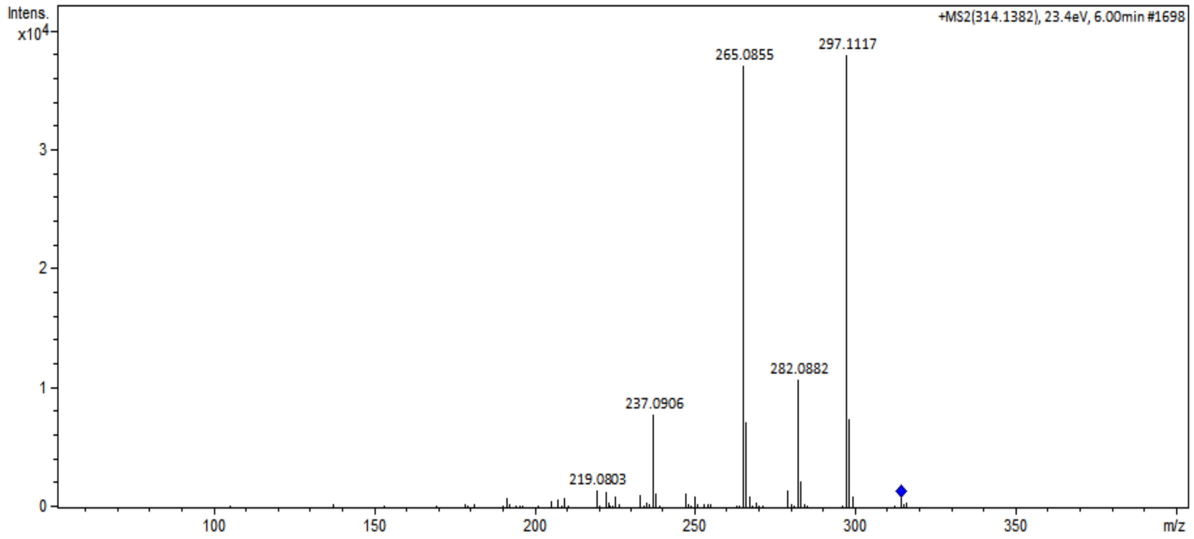

Figure S32: Mass spectra of the feruloyltiramine *m/z* 314.1384 [M+H]⁺ detected in the leaf extract.

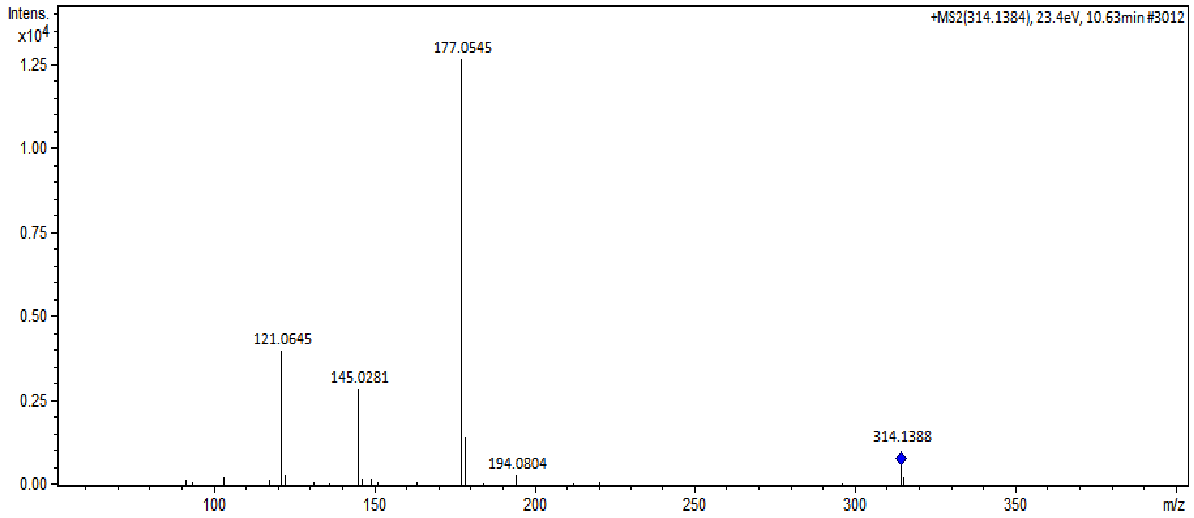

Figure S33: Mass spectra of the *N,O*-dimethylcoclaurine *m/z* 314.1748 [M+H]⁺ detected in the leaf and branch extracts.

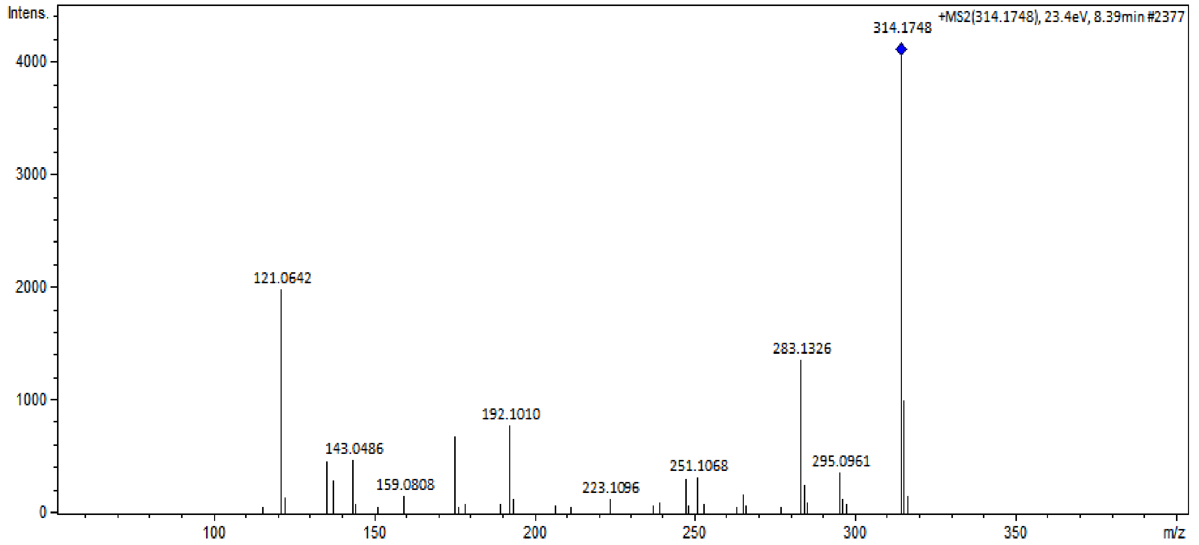

Figure S34: Mass spectra of the dehydrohistidyltryptophyldiketopiperazine *m/z* 322.2725 [M+H]⁺ detected in the leaf and branch extracts.

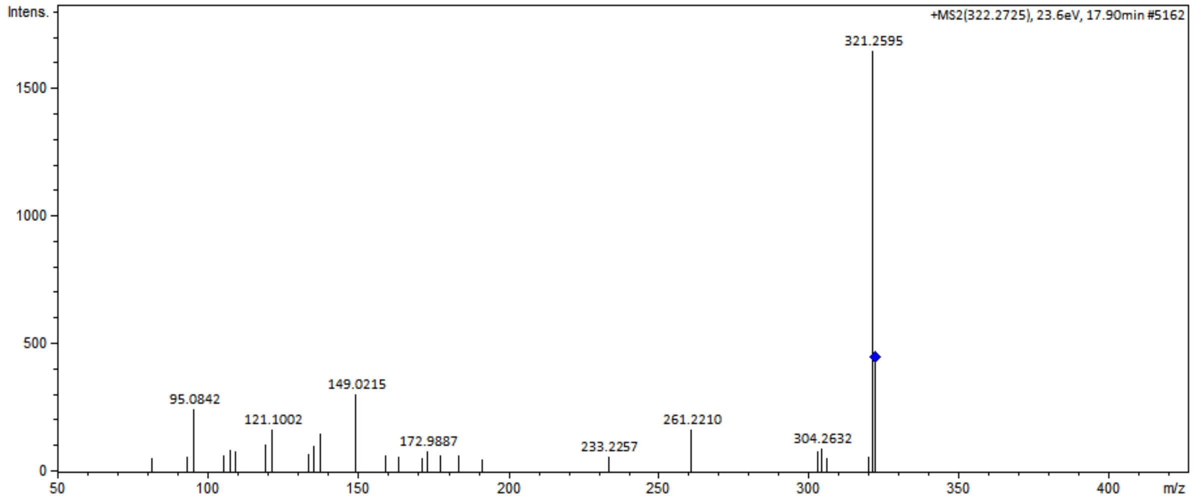

Figure S35: Mass spectra of the boldine *m/z* 328.1541 [M+H]+ detected in the branch extracts.

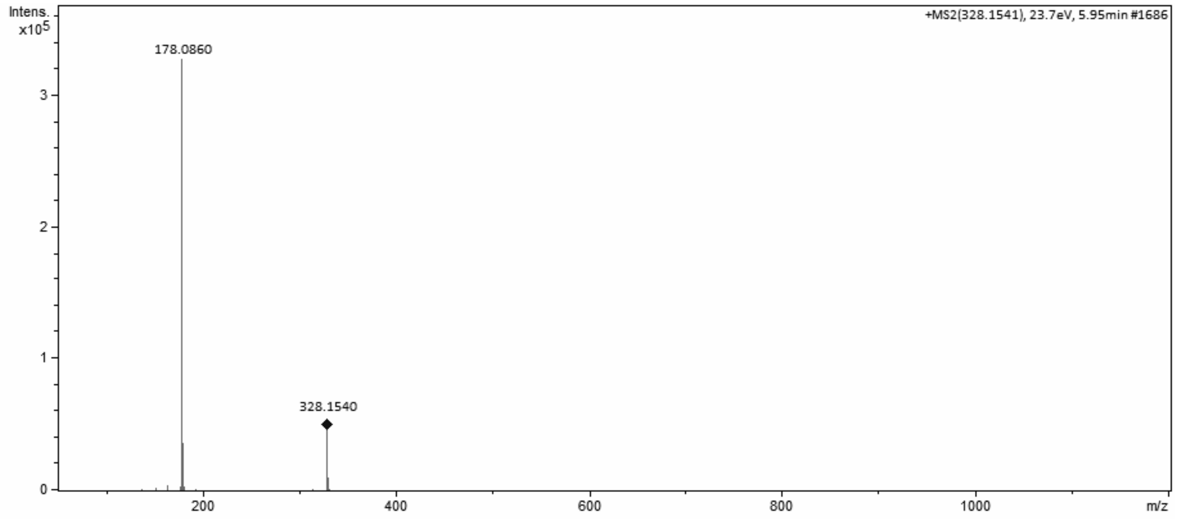

Figure S36: Mass spectra of the reticuline *m/z* 330.0952 [M+H]+ detected in the fungal extracts.

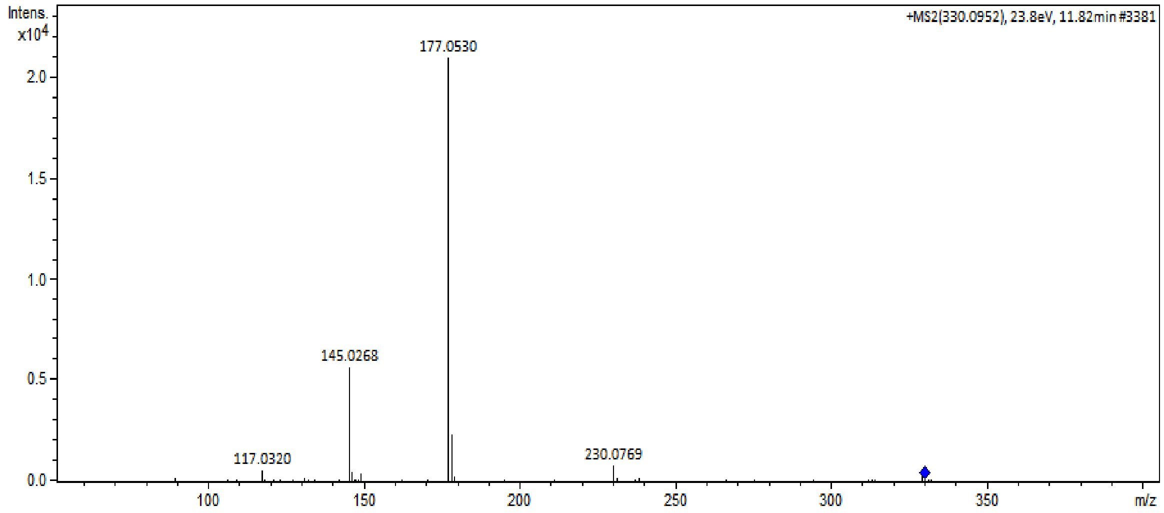

Figure S37: Mass spectra of the *N*-*trans*-feruloyloctopamine *m/z* 330.1331 [M+H]+ detected in the branch extracts.

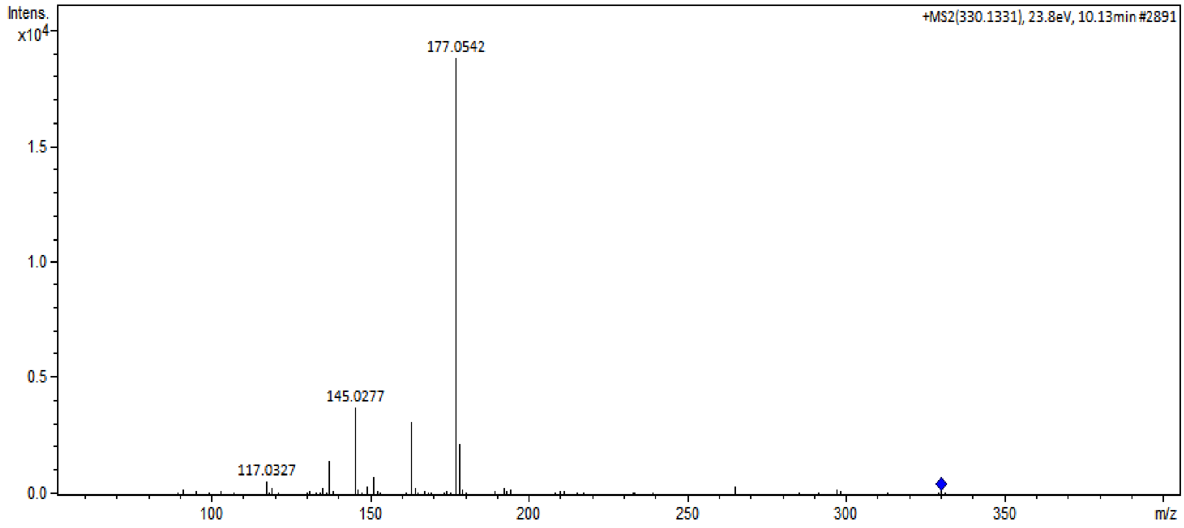

Figure S38: Mass spectra of the reticuline *m/z* 330.1696 [M+H]+ detected in the branch extracts.

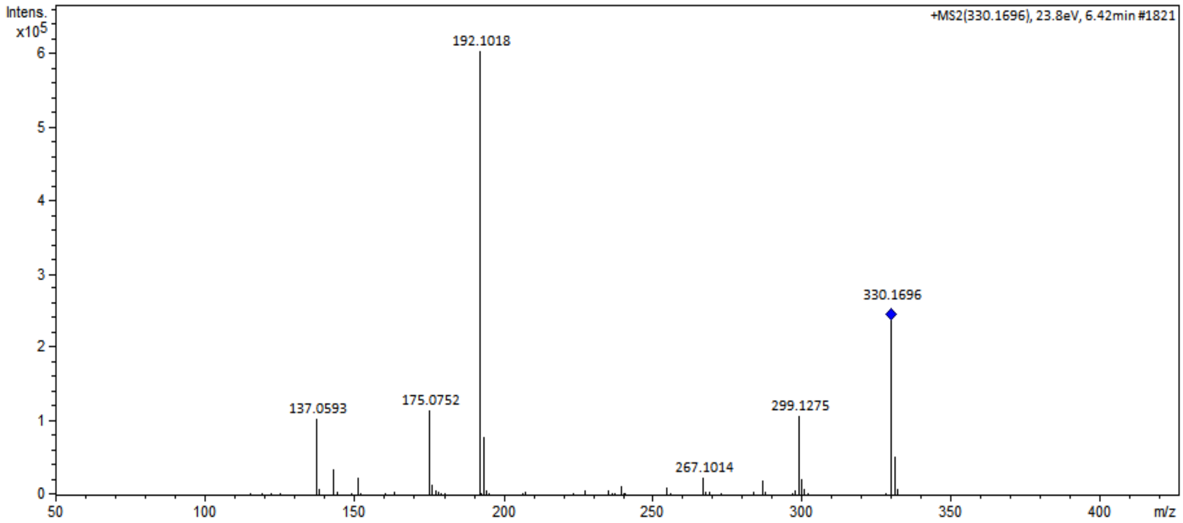

Figure S39: Mass spectra of the crebanine *m/z* 340.1538 [M+H]+ detected in the branch extracts.

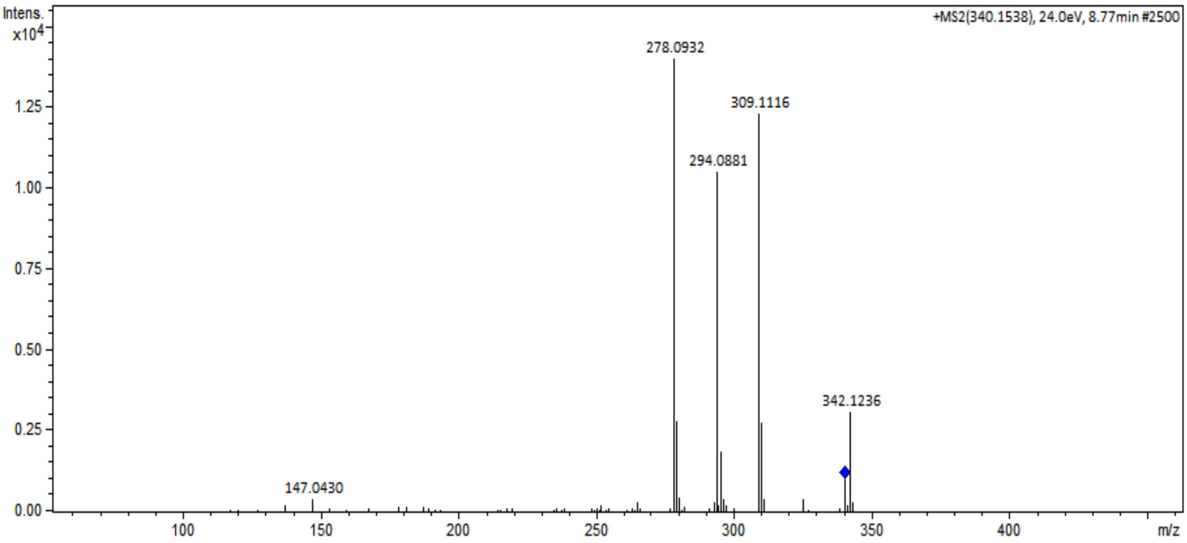

Figure S40: Mass spectra of the isocorydine *m/z* 342.1696 [M+H]+ detected in the leaf and branch extracts.

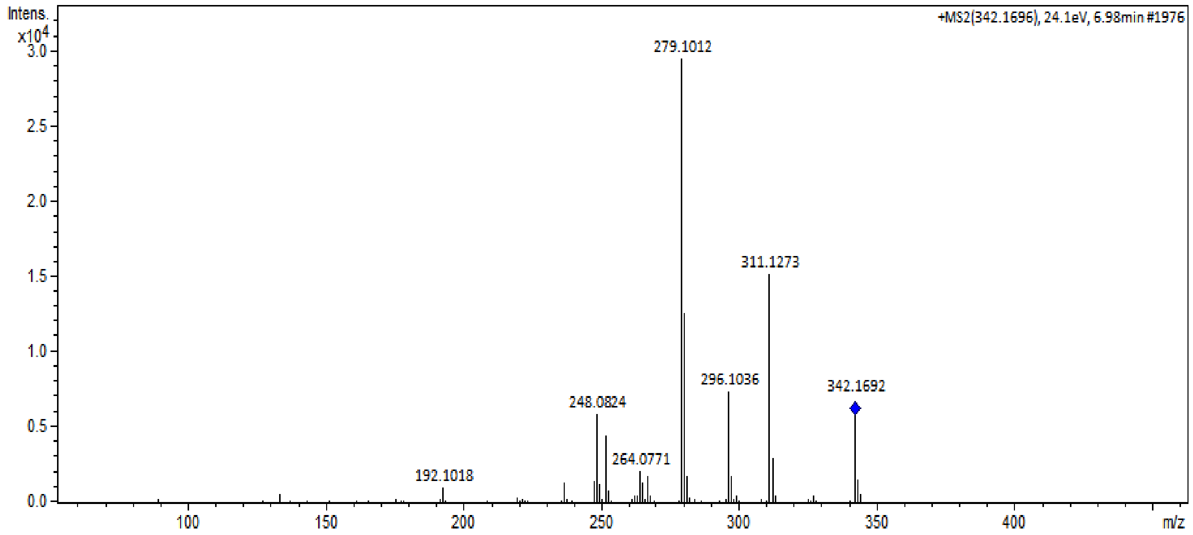

Figure S41: Mass spectra of the norglaucine m/z 342.1698 [M+H]$^+$ detected in the branch extracts.

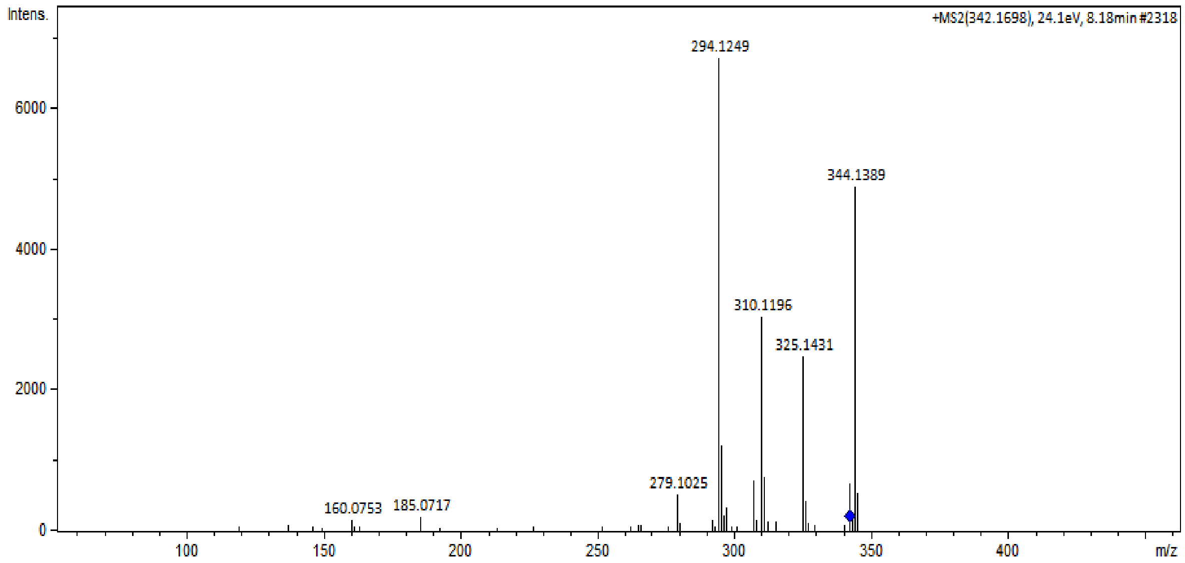

Figure S42: Mass spectra of the *N*-methylaurotetanine m/z 342.1698 [M+H]$^+$ detected in the leaf and branch extracts.

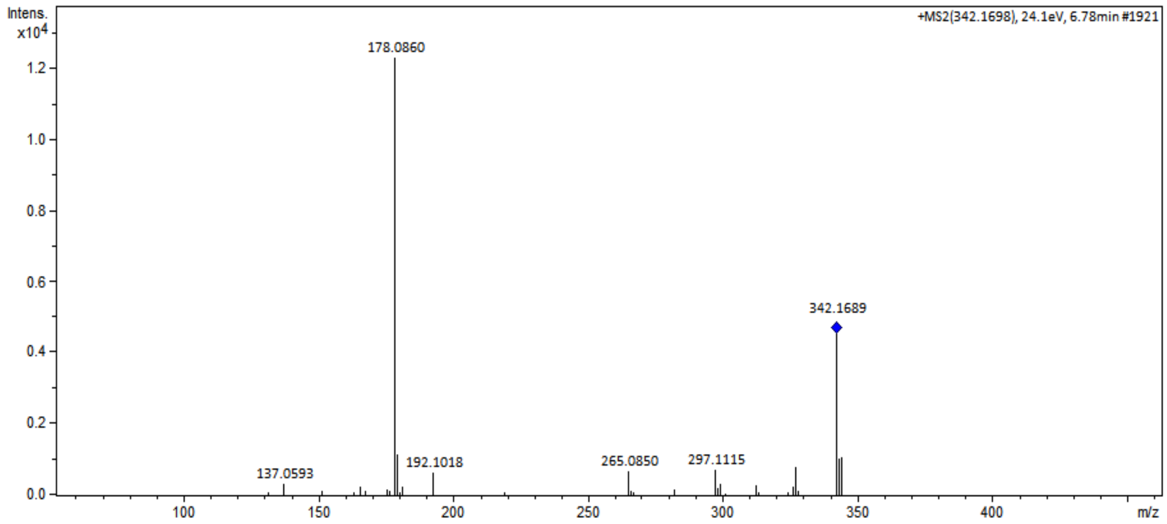

Figure S43: Mass spectra of the 2,3-bis[(4-hydroxy-3-methoxyphenyl)methyl]butane-1,4-diol *m/z* 345.1694 [M-H_2O+H]$^+$ detected in the branch extracts.

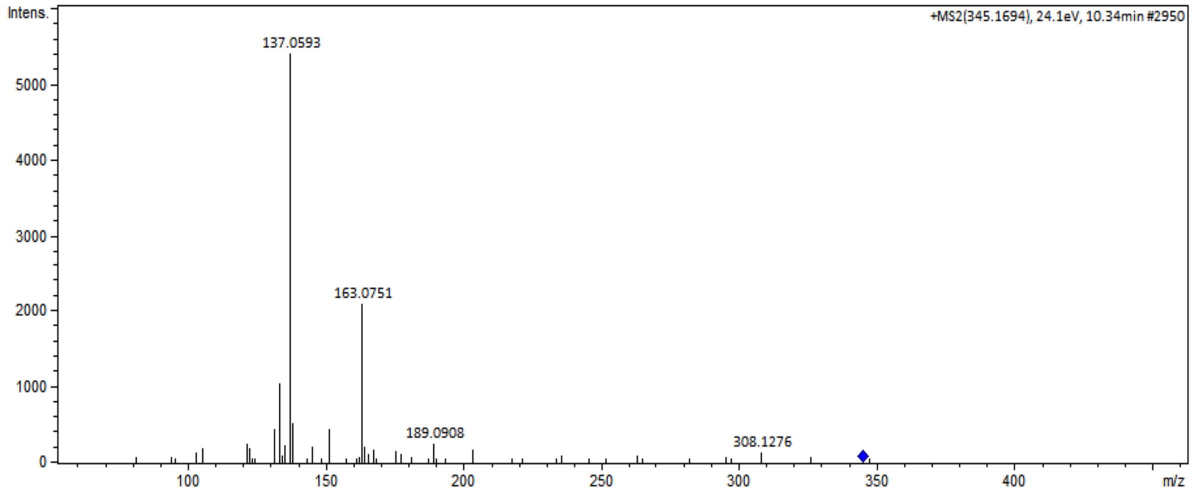

Figure S44: Mass spectra of the chlorogenic acid *m/z* 355.1019 [M+H]$^+$ detected in the branch extracts.

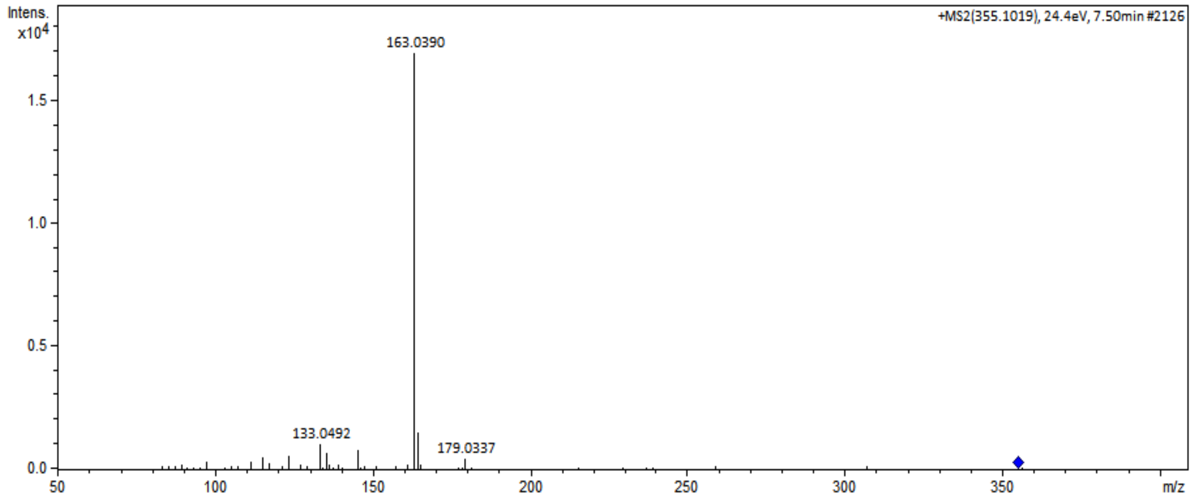

Figure S45: Mass spectra of the glaucine *m/z* 356.1854 [M+H]$^+$ detected in the leaf and branch extracts.

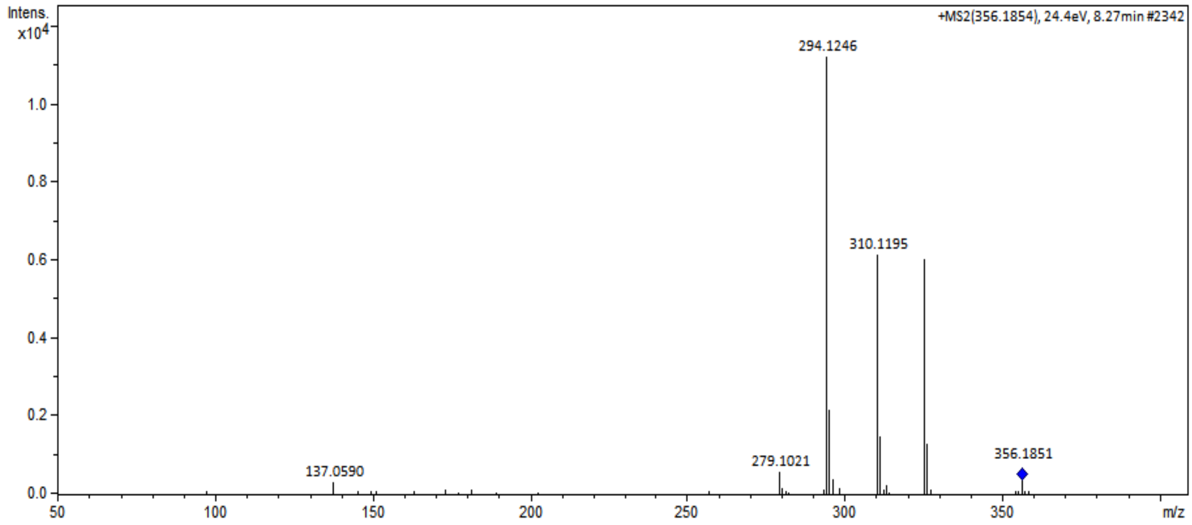

Figure S46: Mass spectra of the (6aS)-1,2,10-trimethoxy-6-methyl-6-oxido-5,6,6a,7-tetrahydro-4H-dibenzo[de,g]quinoline-6-ium-9-ol *m/z* 358.1644 [M+H]$^+$ detected in the leaf and branch extracts.

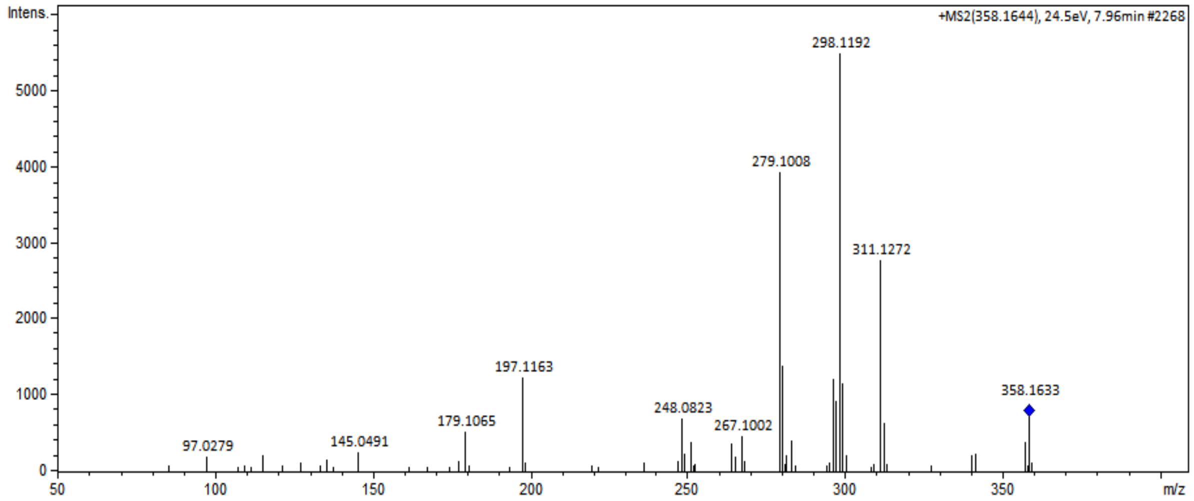

Figure S47: Mass spectra of the fusaperazine E *m/z* 361.1572 [M+H]$^+$ detected in the fungal extracts.

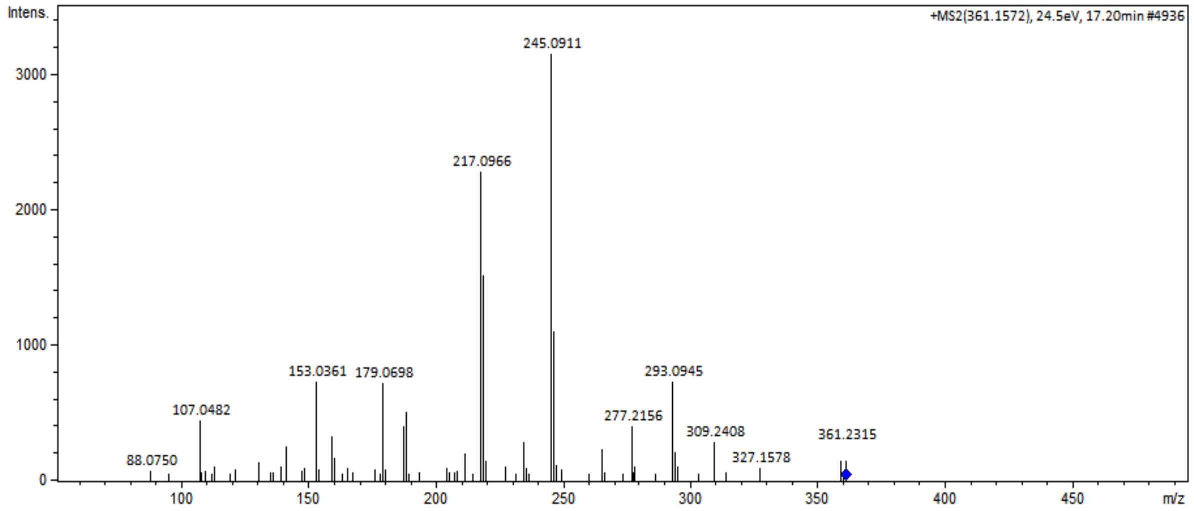

Figure S48: Mass spectra of the 4-(2,6,6-Trimethyl-4-oxo-2-cyclohexen-1-yl)-2-butanylbeta-D-glucopyranoside *m/z* 373.2217 [M+H]$^+$ detected in the leaf and branch extracts.

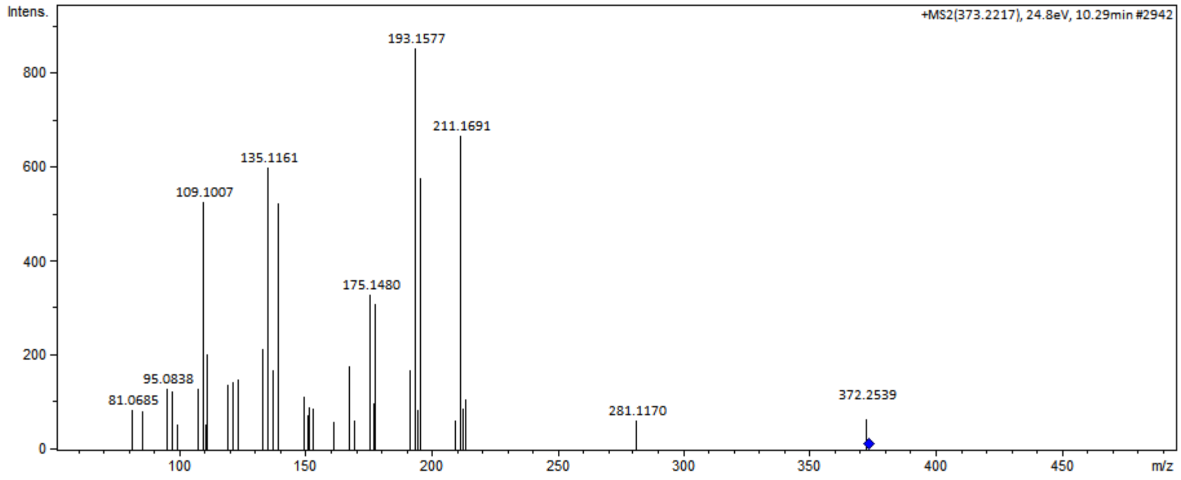

Figure S49: Mass spectra of the (4S)-4-hydroxy-3,5,5-trimethyl-4-[(E)-3-[(2R,3R,4S,5S,6R)-3,4,5-trihydroxy-6-(hydroxymethyl)oxan-2-yl]oxybut-1-enyl]cyclohex-2-en-1-one *m/z* 387.2004[M+H]$^+$ detected in the leaf extract.

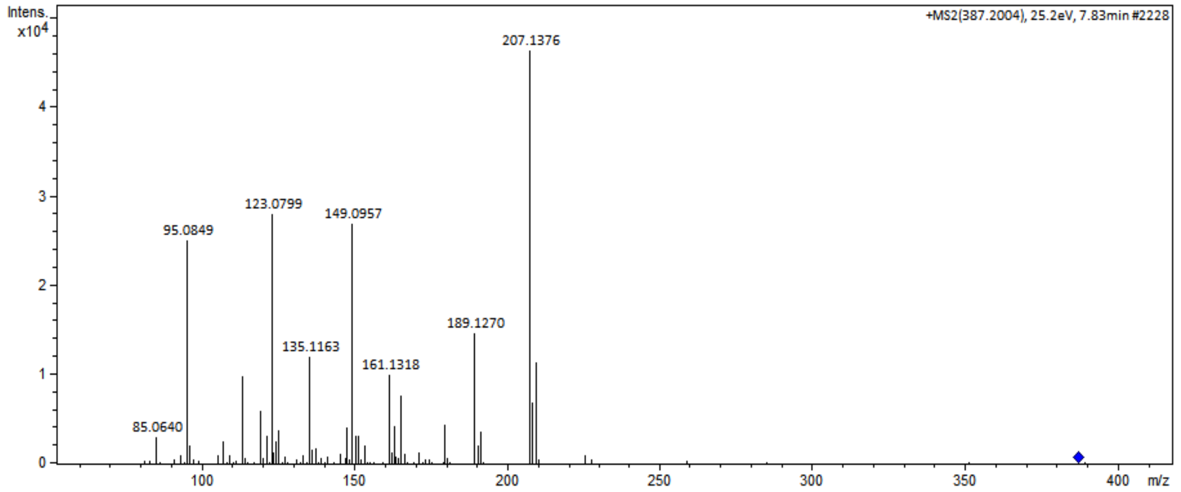

Figure S50: Mass spectra of the roquefortine C *m/z* 390.1929 $[M+H]^+$ detected in the fungal extracts.

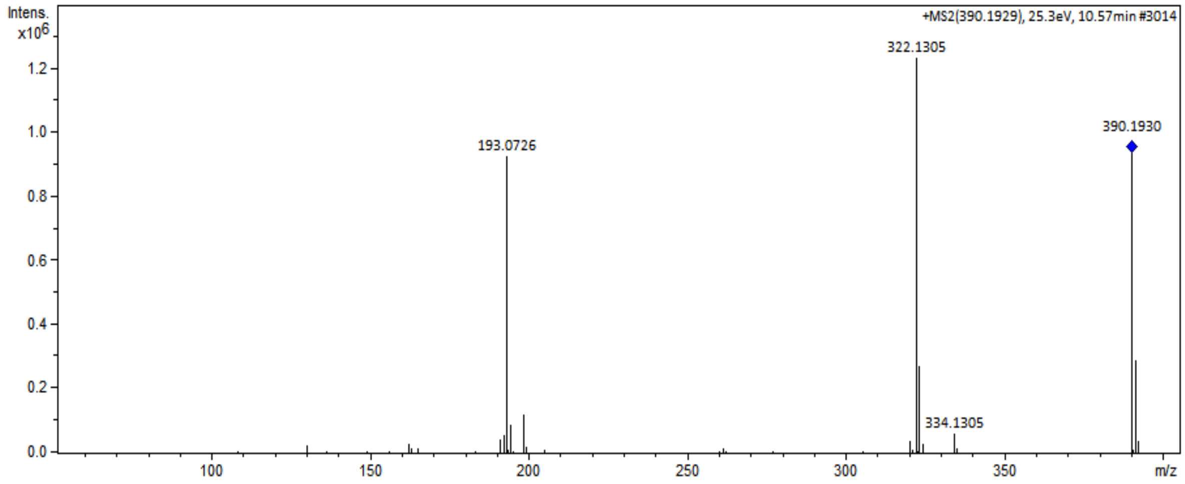

Figure S51: Mass spectra of the roquefortine D *m/z* 392.2081 $[M+H]^+$ detected in the fungal extracts.

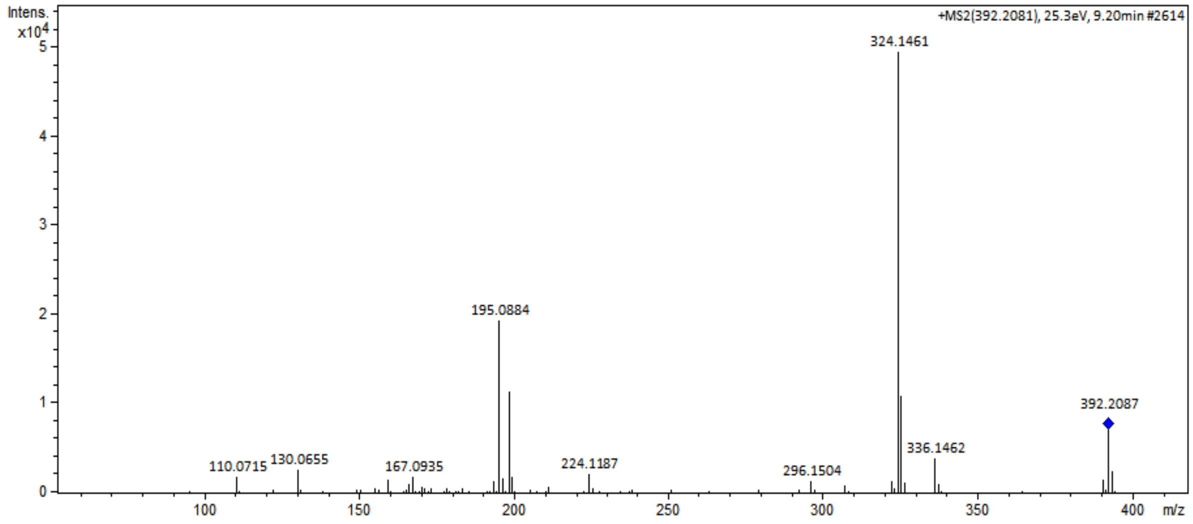

Figure S52: Mass spectra of the Hydroxy-roquefortine C *m/z* 406.1871 $[M+H]^+$ detected in the fungal extracts.

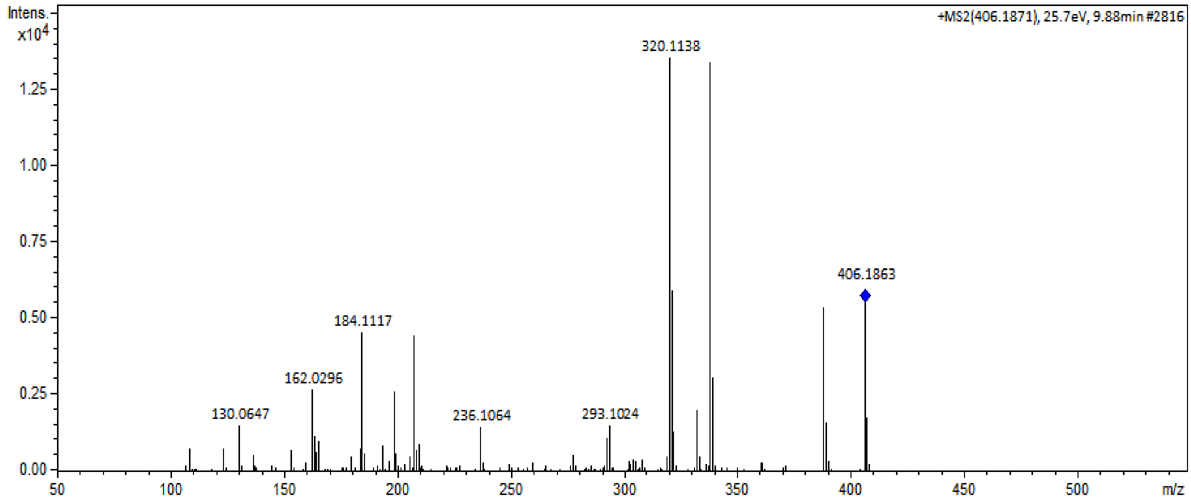

Figure S53: Mass spectra of the syringaresinol *m/z* 419.1678 [M+H]$^+$ detected in the branch extract.

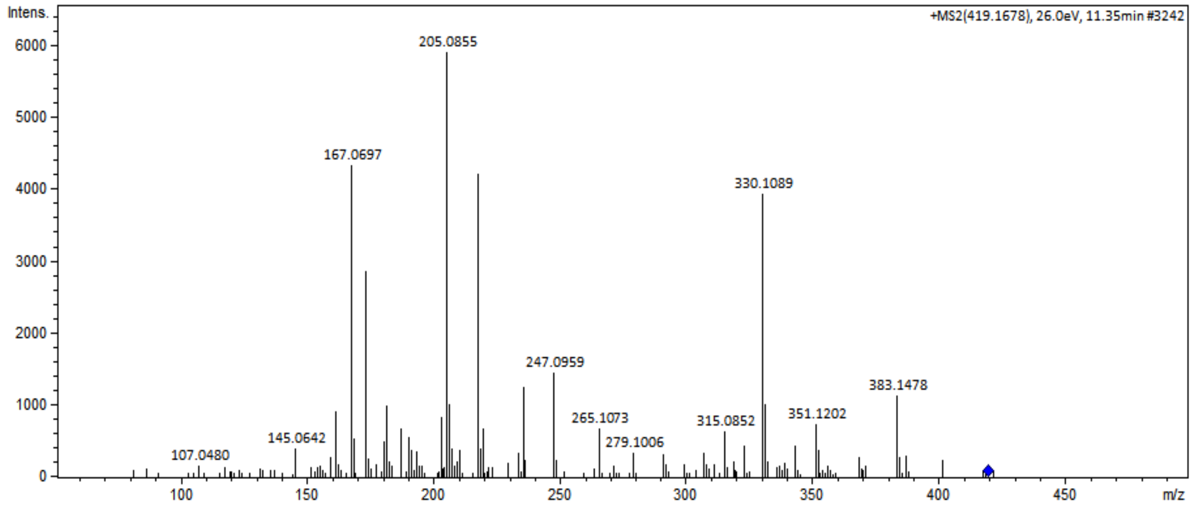

Figure S54: Mass spectra of the mangiferin *m/z* 423.0919 [M+H]$^+$ detected in the leaf and branch extracts.

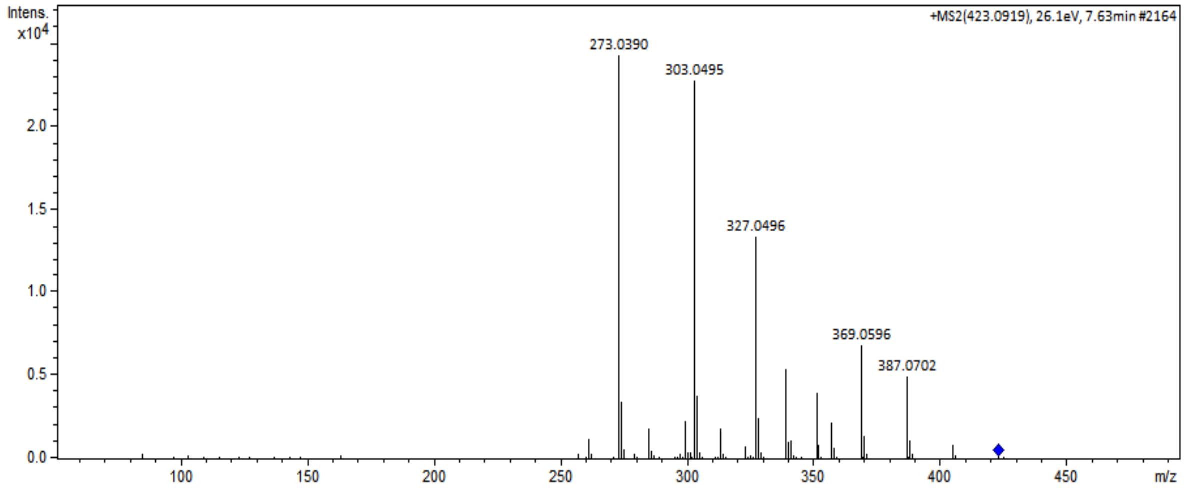

Figure S55: Mass spectra of the astragalin *m/z* 449.1070 [M+H]$^+$ detected in the leaf extract.

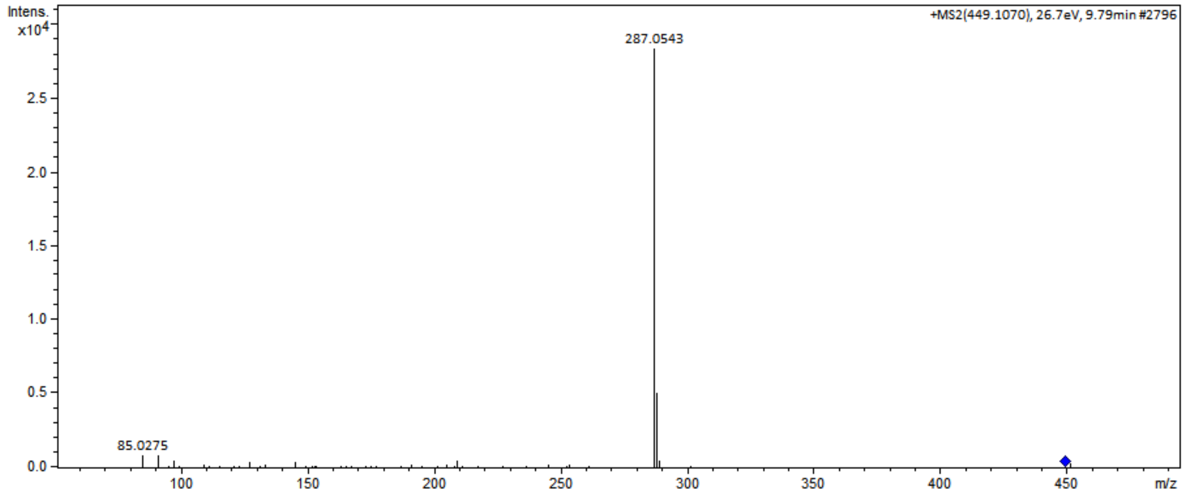

Figure S56: Mass spectra of the isoquercetin *m/z* 465.1029 $[M+H]^+$ detected in the leaf and branch extract.

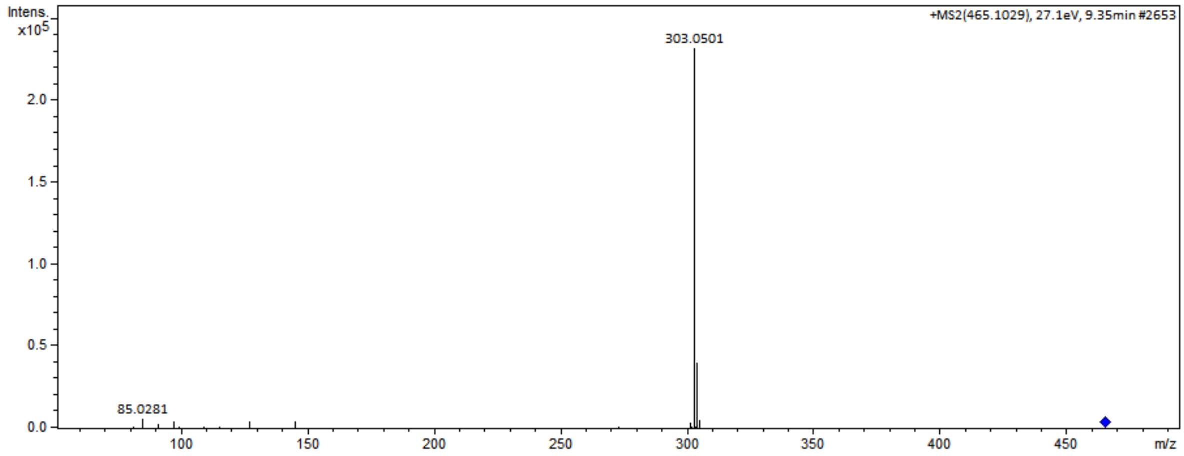

Figure S57: Mass spectra of the procyanidin B2 *m/z* 579.1491 $[M+H]^+$ detected in the branch extract.

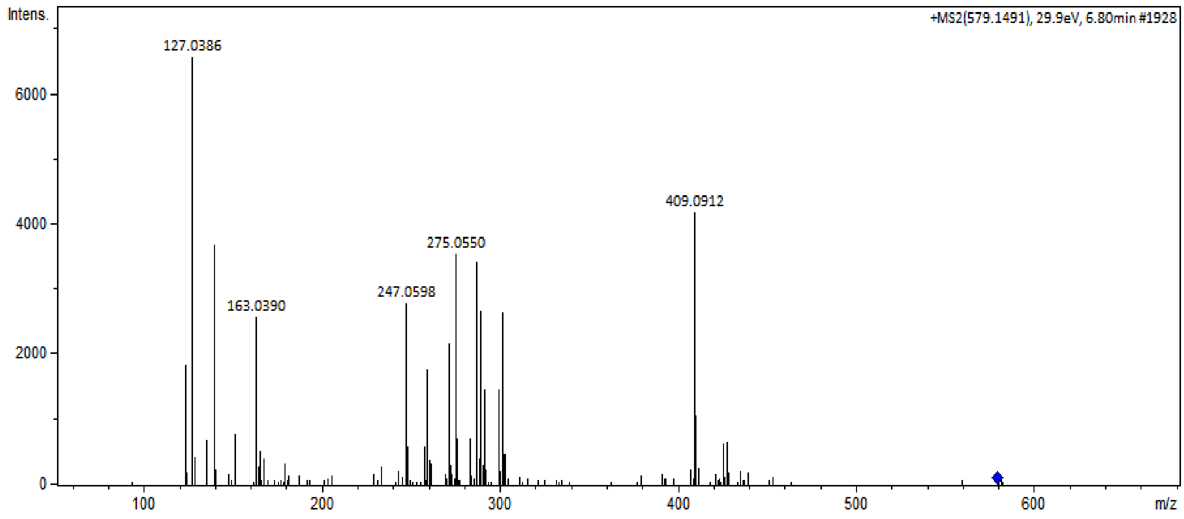

Figure S58: Mass spectra of the tiliroside *m/z* 595.1434 $[M+H]^+$ detected in the leaf and branch extracts.

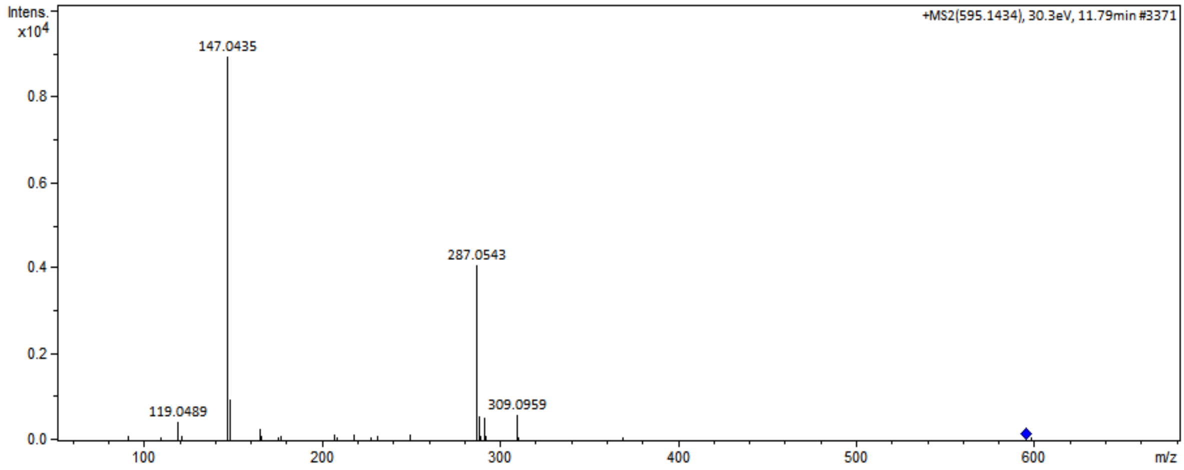

Figure S59: Mass spectra of the kaempferol 3-O-rutinoside *m/z* 595.1651 $[M+H]^+$ detected in the leaf and branch extracts.

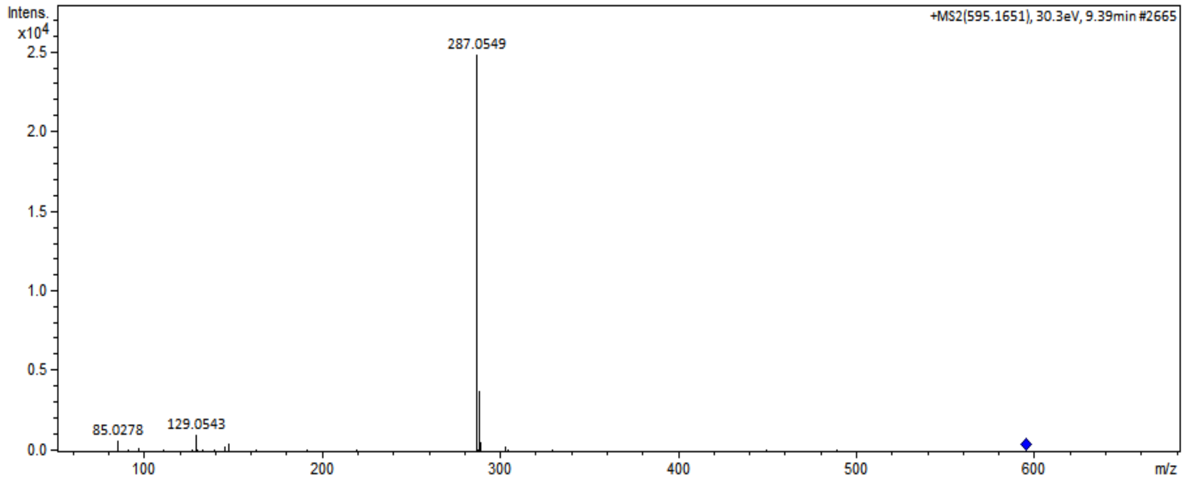

Figure S60: Mass spectra of the quercetin-3-O-vicianoside *m/z* 597.1433 $[M+H]^+$ detected in the leaf and branch extracts.

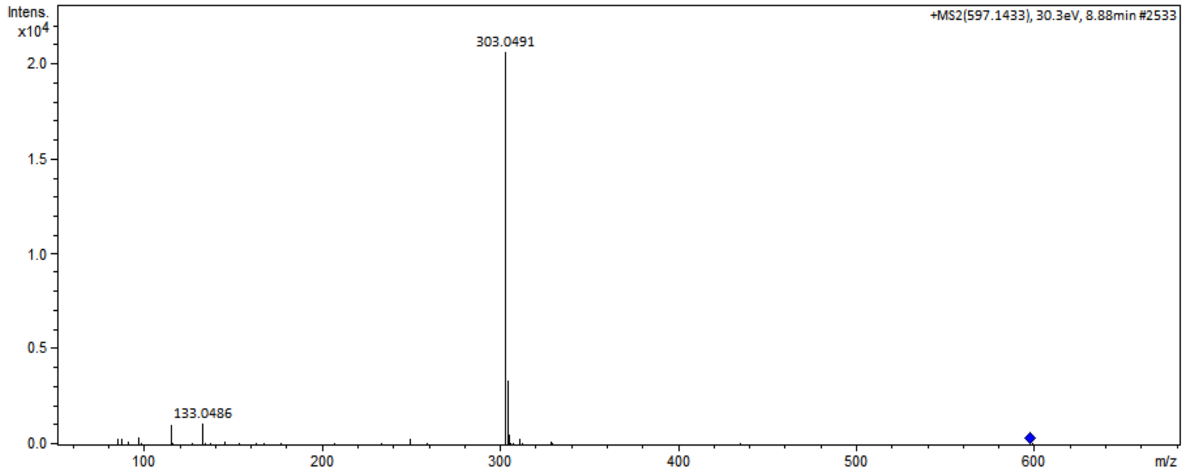

Figure S61: Mass spectra of the rutin *m/z* 611.1598 $[M+H]^+$ detected in the leaf and branch extracts.

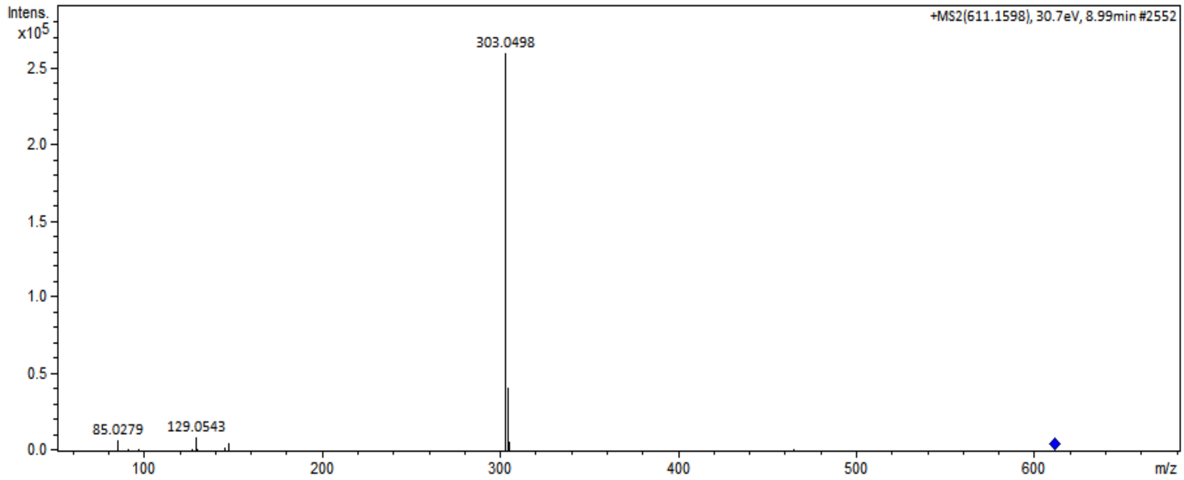

Figure S62: Mass spectra of the quercetin 3 *m/z* 743.2012 $[M+H]^+$ detected in the branch extract.

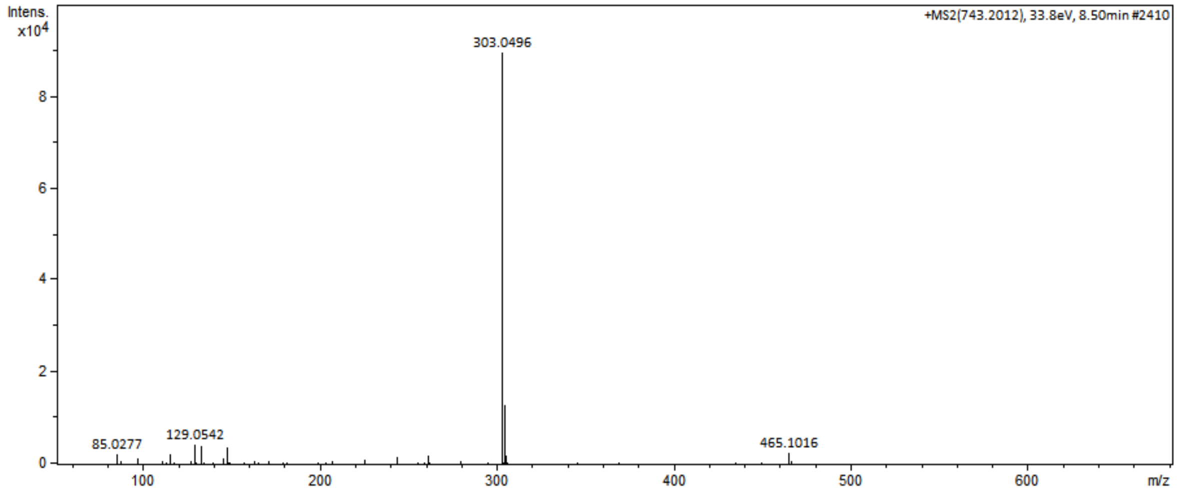

Table S1: Secondary metabolites putatively identified in fungal extracts and plant extract

	Assigned compounds	Molecular Formula	Mass measured	Theoretical Mass	Adduct
**Branch extract**					
**1**	Loliolide	$C_{11}H_{16}O_3$	197.1168	197.1172	$[M+H]^+$
**2**	3,5,7,2',6'-pentahydroxyflavanone	$C_{15}H_{12}O_7$	305.0652	305.0658	$[M+H]^+$
**3**	Boldine	$C_{19}H_{21}NO_4$	328.1541	328.1543	$[M+H]^+$
**4**	Reticuline	$C_{19}H_{23}NO_4$	330.1696	330.1699	$[M+H]^+$
**5**	Crebanine	$C_{20}H_{21}NO_4$	340.1538	340.1543	$[M+H]^+$
**6**	2,3-bis[(4-hydroxy-3-methoxyphenyl)methyl]butane-1,4-diol	$C_{20}H_{26}O_6$	345.1694	345.1702	$[M-H_2O+H]^+$
**7**	Chlorogenic Acid	$C_{16}H_{18}O_9$	355.1019	355.1023	$[M+H]^+$
**8**	Syringaresinol	$C_{22}H_{26}O_8$	419.1678	419.1700	$[M+H]^+$
**9**	Procyanidin B2	$C_{30}H_{26}O_{12}$	579.1491	579.1497	$[M+H]^+$
**10**	Quercetin 3	$C_{32}H_{38}O_{20}$	743.2012	743.2029	$[M+H]^+$
**Leaf extract**					
**11**	Feruloyltyramine	$C_{18}H_{19}NO_4$	314.1384	314.1387	$[M+H]^+$

**12**	(4S)-4-hydroxy-3,5,5-trimethyl-4-[(E)-3-[(2R,3R,4S,5S,6R)-3,4,5-trihydroxy-6-(hydroxymethyl)oxan-2-yl]oxybut-1-enyl]cyclohex-2-en-1-one	$C_{19}H_{30}O_8$	387.2004	387.2014	$[M+H]^+$
**13**	Astragalin	$C_{21}H_{20}O_{11}$	449.1070	449.1078	$[M+H]^+$
			**Leaf and branch extracts**		
**14**	3-Hydroxy-4-methoxycinnamic acid	$C_{10}H_{10}O_4$	177.0543	177.0552	$[M-H_2O+H]^+$
**15**	Alismol	$C_{15}H_{24}O$	203.1790	203.1799	$[M-H_2O+H]^+$
**16**	alpha-Bisabolol	$C_{15}H_{26}O$	205.0813	205.1950	$[M-H_2O+H]^+$
**17**	Jasmonic Acid	$C_{12}H_{18}O_3$	211.1324	211.1328	$[M+H]^+$
**18**	Zedoarondiol	$C_{15}H_{24}O_3$	235.1690	235.1692	$[M-H_2O+H]^+$
**19**	Asimilobine	$C_{17}H_{17}NO_2$	268.1324	268.1332	$[M+H]^+$
**20**	Naringenin	$C_{15}H_{12}O_5$	273.0753	273.0757	$[M+H]^+$
**21**	Paprazine	$C_{17}H_{17}NO_3$	284.1280	284.1281	$[M+H]^+$
**22**	Coclaurine	$C_{17}H_{19}NO_3$	286.1434	286.1438	$[M+H]^+$
**23**	Eriodictiol	$C_{15}H_{12}O_6$	289.0705	289.0707	$[M+H]^+$
**24**	Epicatechin	$C_{15}H_{14}O_6$	291.0860	291.0863	$[M+H]^+$
**25**	Stepharine	$C_{18}H_{19}NO_3$	298.1435	298.1438	$[M+H]^+$
**26**	N-Methylcoclaurine	$C_{18}H_{21}NO_3$	300.1594	300.1594	$[M+H]^+$

**27**	Norboldine	$C_{18}H_{19}NO_4$	314.1382	314.1387	$[M+H]^+$
**28**	N,O-Demethylcoclaurine	$C_{19}H_{23}NO_3$	314.1748	314.1751	$[M+H]^+$
**29**	N-trans-Feruloyloctopamine	$C_{18}H_{19}NO_5$	330.1331	330.1336	$[M+H]^+$
**30**	Isocorydine	$C_{20}H_{23}NO_4$	342.1696	342.1699	$[M+H]^+$
**31**	Norglaucine	$C_{20}H_{23}NO_4$	342.1698	342.1699	$[M+H]^+$
**32**	N-methylaurotetanine	$C_{20}H_{23}NO_4$	342.1698	342.1699	$[M+H]^+$
**33**	Glaucine	$C_{21}H_{25}NO_4$	356.1854	356.1856	$[M+H]^+$
**34**	(6aS)-1,2,10-trimethoxy-6-methyl-6-oxido-5,6,6a,7-tetrahydro-4H-dibenzo[de,g]quinoline-6-ium-9-ol	$C_{20}H_{23}NO_5$	358.1644	358.1649	$[M+H]^+$
**35**	4-(2,6,6-Trimethyl-4-oxo-2-cyclohexen-1-yl)-2-butanylbeta-D-glucopyranoside	$C_{19}H_{32}O_7$	373.2217	373.2221	$[M+H]^+$
**36**	Mangiferin	$C_{19}H_{18}O_{11}$	423.0919	423.0922	$[M+H]^+$
**37**	Isoquercetin	$C_{21}H_{20}O_{12}$	465.1029	465.1027	$[M+H]^+$
**38**	Tiliroside	$C_{30}H_{26}O_{13}$	595.1434	595.1446	$[M+H]^+$
**39**	Kaempferol 3-O-rutinoside	$C_{27}H_{30}O_{15}$	595.1651	595.1657	$[M+H]^+$

**40**	Quercetin-3-O-vicianoside	$C_{26}H_{28}O_{16}$	597.1433	597.1450	$[M+H]^+$
**41**	Rutin	$C_{27}H_{30}O_{16}$	611.1598	611.1606	$[M+H]^+$
		**Fungal and plant extract**			
**42**	Coumaryl acetate	$C_{11}H_{12}O_3$	193.0701	193.0859	$[M+H]^+$
**43**	Valerianol	$C_{15}H_{26}O$	223.1680	223.2056	$[M+H]^+$
**44**	Sinapic acid	$C_{11}H_{12}O_5$	225.0750	225.0757	$[M+H]^+$
**45**	Canangalia H	$C_{16}H_{28}O_4$	249.1843	249.1848	$[M-2H_2O+H]^+$
		**Fungal extracts**			
**46**	Vanillic acid	$C_8H_8O_4$	169.0968	169.0495	$[M+H]^+$
**47**	Gamma-Decalactone	$C_{10}H_{18}O_2$	171.1374	171.1379	$[M+H]^+$
**48**	p-Acetaminobenzoic acid	$C_9H_9NO_3$	180.0649	180.0655	$[M+H]^+$
**49**	Viridicatin	$C_{15}H_{11}NO_2$	238.0869	238.0863	$[M+H]^+$
**50**	Viridicatol	$C_{15}H_{11}NO_3$	254.0815	254.0812	$[M+H]^+$
**51**	4-phenyl-3,4-dihydroquinolin-2(1H)-one	$C_{15}H_{13}NO_3$	256.0971	256.0968	$[M+H]^+$

**52**	Cyclopeptin	$C_{17}H_{16}N_2O_2$	281.1285	281.1284	$[M+H]^+$
**53**	Dehydrocurvularin	$C_{16}H_{18}O_5$	291.1213	291.1227	$[M+H]^+$
**54**	Curvularin	$C_{16}H_{20}O_5$	293.1367	293.1383	$[M+H]^+$
**55**	Cyclopenin	$C_{17}H_{14}N_2O_3$	295.1080	295.1077	$[M+H]^+$
**56**	Cyclopenol	$C_{17}H_{14}N_2O_4$	311.1032	311.1026	$[M+H]^+$
**57**	Dehydrohistidyltryptophyldiketopiperazine	$C_{17}H_{15}N_5O_2$	322.2725	322.1299	$[M+H]^+$
**58**	Avenanthramide B	$C_{17}H_{15}NO_6$	330.0952	330.0972	$[M+H]^+$
**59**	Fusaperazine E	$C_{19}H_{24}N_2O_3S$	361.1572	361.1572	$[M+H]^+$
**60**	Roquefortine C	$C_{22}H_{23}N_5O_2$	390.1929	390.1924	$[M+H]^+$
**61**	Roquefortine D	$C_{22}H_{25}N_5O_2$	392.2081	392.2081	$[M+H]^+$
**62**	Hydroxy-roquefortine C	$C_{22}H_{23}N_5O_3$	406.1871	406.1873	$[M+H]^+$
